# Data-Driven Analysis of Age, Sex, and Tissue Effects on Gene Expression Variability in Alzheimer's Disease

**DOI:** 10.3389/fnins.2019.00392

**Published:** 2019-04-24

**Authors:** Lavida R. K. Brooks, George I. Mias

**Affiliations:** ^1^Microbiology and Molecular Genetics, Institute for Quantitative Health Science and Engineering, Michigan State University, East Lansing, MI, United States; ^2^Biochemistry and Molecular Biology, Institute for Quantitative Health Science and Engineering, Michigan State University, East Lansing, MI, United States

**Keywords:** Alzheimer's disease, neurodegeneration, transcriptomics, meta-analysis, microarray analysis, aging, bioinformatics

## Abstract

Alzheimer's disease (AD) has been categorized by the Centers for Disease Control and Prevention (CDC) as the 6^th^ leading cause of death in the United States. AD is a significant health-care burden because of its increased occurrence (specifically in the elderly population), and the lack of effective treatments and preventive methods. With an increase in life expectancy, the CDC expects AD cases to rise to 15 million by 2060. Aging has been previously associated with susceptibility to AD, and there are ongoing efforts to effectively differentiate between normal and AD age-related brain degeneration and memory loss. AD targets neuronal function and can cause neuronal loss due to the buildup of amyloid-beta plaques and intracellular neurofibrillary tangles. Our study aims to identify temporal changes within gene expression profiles of healthy controls and AD subjects. We conducted a meta-analysis using publicly available microarray expression data from AD and healthy cohorts. For our meta-analysis, we selected datasets that reported donor age and gender, and used Affymetrix and Illumina microarray platforms (8 datasets, 2,088 samples). Raw microarray expression data were re-analyzed, and normalized across arrays. We then performed an analysis of variance, using a linear model that incorporated age, tissue type, sex, and disease state as effects, as well as study to account for batch effects, and included binary interactions between factors. Our results identified 3,735 statistically significant (Bonferroni adjusted *p* < 0.05) gene expression differences between AD and healthy controls, which we filtered for biological effect (10% two-tailed quantiles of mean differences between groups) to obtain 352 genes. Interesting pathways identified as enriched comprised of neurodegenerative diseases pathways (including AD), and also mitochondrial translation and dysfunction, synaptic vesicle cycle and GABAergic synapse, and gene ontology terms enrichment in neuronal system, transmission across chemical synapses and mitochondrial translation. Overall our approach allowed us to effectively combine multiple available microarray datasets and identify gene expression differences between AD and healthy individuals including full age and tissue type considerations. Our findings provide potential gene and pathway associations that can be targeted to improve AD diagnostics and potentially treatment or prevention.

## 1. Introduction

Aging refers to the physiological changes that occur within the body overtime (Lopez-Otin et al., 2013). These changes are accompanied by deteriorating cell and organ function due to cellular and immune senescence and DNA and protein damage (Lopez-Otin et al., 2013; Van Deursen, 2014; Childs et al., 2015). Aging causes an increased risk for diseases. Age-related diseases are becoming a public health concern due to an overall increase in the older population and the average human life span in developed countries (Black et al., 2015; Rowe et al., 2016). It is predicted that by the year 2050, the number of Americans over 85 years of age will triple from 2015 (United Nations Department of Economic and Social Affairs, 2015; Jaul and Barron, 2017). Larger percentages of the elderly and their increased risk for diseases can affect the economy, and social and health care costs (Dallmeyer et al., 2017). For instance, immune system dysfunction and cognitive decline due to aging increases the risk of neurodegenerative diseases, such as Alzheimer's disease (AD) (Jevtic et al., 2017; Mattson and Arumugam, 2018). Previous research explored brain aging and found notable changes in brain size, brain structure and function (Drayer, 1988). Changes in the brain as we age are also known as hallmarks of brain aging. These hallmarks include: mitochondrial dysfunction, damage to proteins and DNA due to oxidation, neuroinflammation due to immune system dysfunction, reduction in brain volume size and gray and white matter, and impaired regulation of neuronal Ca^2+^ (Drayer, 1988; Mattson and Arumugam, 2018). These alterations render the aging brain vulnerable to neurodegenerative diseases, such as AD.

AD, the most common form of dementia, is currently the 6^th^ leading cause of death (Taylor et al., 2017) in the United States (US). In 2010, an estimate of 4.7 million people in the US had AD, and the number of AD patients is expected to increase to 13.8 million in 2050 and to 15 million by 2060 (Hebert et al., 2013; Brookmeyer et al., 2018; Matthews et al., 2018). As with other age-related diseases, the risk of AD increases with age. AD is currently characterized by the accumulation of amyloid-beta (Aβ) plaques and neurofibrillary tangles due to tau protein modifications (Masters et al., 2015). These two protein changes are the main pathological changes in AD (Masters et al., 2015). Aβ is formed when the amyloid precursor protein (APP) is cleaved by γ-secretases and β-secretases. Cleavage of APP forms fragments of Aβ which aggregate and deposit on neurons as plaques, which causes neuronal death in conjunction with neurofibrillary tangles (Masters et al., 2015).

While AD's prevalence is on the rise due to increased life expectancy, there is still no treatment available and diagnosis of AD is challenging. How AD progresses is still not completely understood (De Jager et al., 2018). New technologies are available, such as positron-emission tomography (PET) imaging and monitoring levels of Aβ and tau in cerebrospinal fluid (Masters et al., 2015). Co-morbidities that can exist due to aging, such as hippocampal sclerosis further complicate AD diagnosis (Toepper, 2017). Furthermore, questions have been raised regarding whether or not AD is simply an accelerated form of aging due to them both being associated with changes in cognition (Toepper, 2017). However, studies have identified clear neurocognitive differences in cognition, brain size and function in AD compared to healthy aged subjects. For example, AD patients have more gray matter loss compared to white matter, impaired verbal and semantic abilities and more intense memory dysfunction compared to healthy seniors (Toepper, 2017).

Pathological changes within the brain are observed prior to clinical diagnosis of AD. In most cases AD cannot be confirmed until postmortem examination of the brain. Researchers are investigating novel biomarkers to detect for earlier diagnosis before diseased individuals become functionally impaired. Meta-analysis of microarray datasets is becoming more popular for it provides stronger power to studies due to larger sample sizes obtained through statistically combining multiple datasets. Microarray data are also available in large quantities on public online data repositories. In the case of AD, Winkler and Fox performed a meta-analysis that compared neurons within the hippocampus of AD patients and healthy controls. They identified that processes, such as apoptosis, and protein synthesis, were affected by AD and were regulated by androgen and estrogen receptors (Winkler and Fox, 2013). Researchers have also explored differences in gene expression in Parkinson's and AD subjects via a meta-analysis approach (Wang et al., 2017), and identified functionally enriched genes and pathways that showed overlap between the two diseases (Wang et al., 2017). Most recently, Moradifard et al. identified differentially expressed microRNAs and genes when comparing AD to healthy controls via a meta-analysis approach. They also identified two key microRNAs that act as regulators in the AD gene network (Moradifard et al., 2018).

In our investigation, our goal was to identify age, sex, and tissue effects on gene expression variability in AD by comparing age-matched healthy controls to AD subjects via a meta-analysis approach. In this data-driven approach, we explored global gene expression changes in 2,088 total samples (771 healthy, 868 AD, and 449 possible AD, curated from eight studies) from 26 different tissues, to identify genes and pathways of interest in AD that can be affected by factors, such as age, sex, and tissue. Our findings provide potential gene and pathway associations that can be targeted to improve AD diagnostics and potentially treatment or prevention.

## 2. Methods

**Figure 1:**
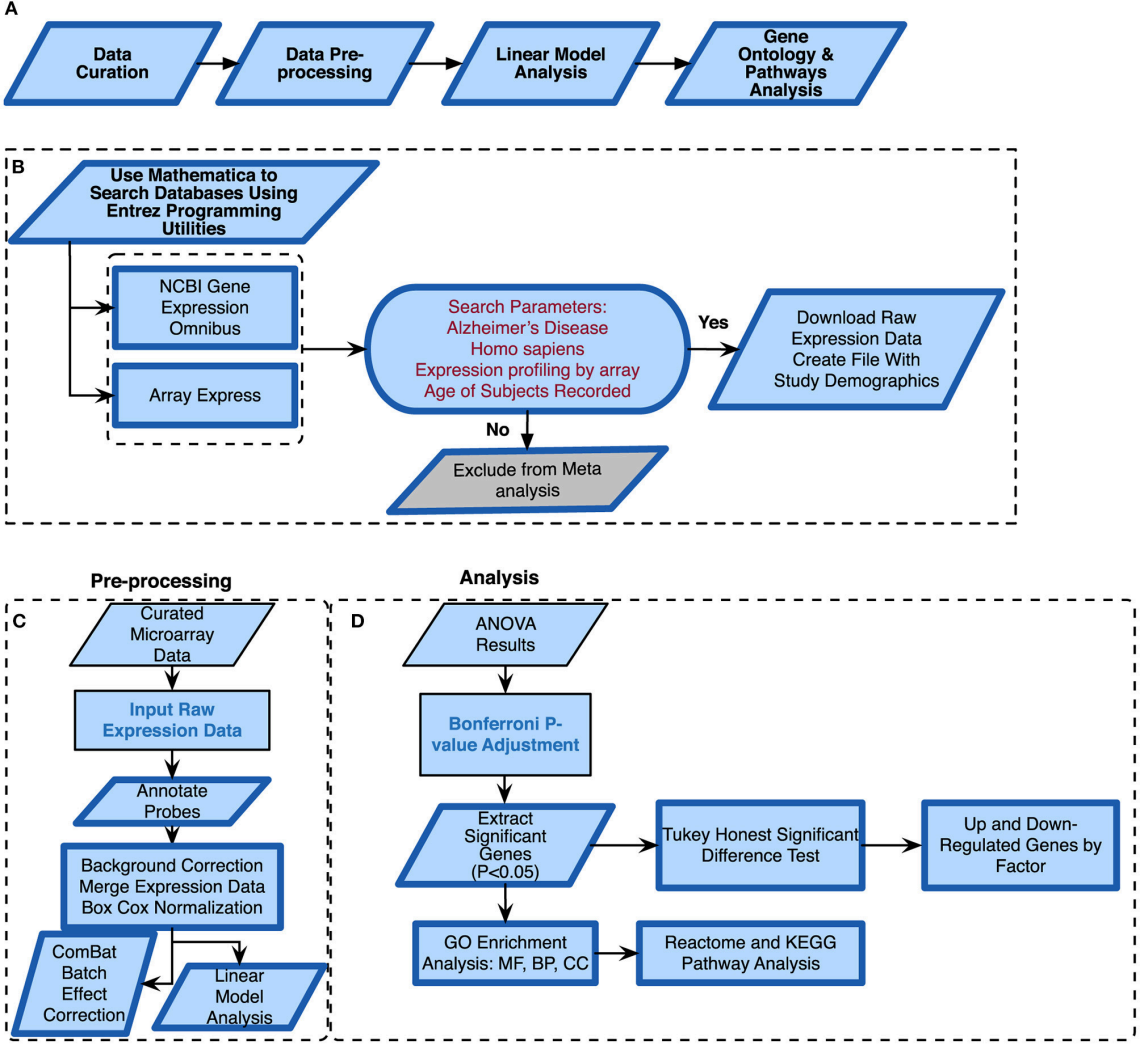
Alzheimer's disease meta-analysis framework. **(A)** Simplified workflow used for the meta-analysis, **(B)** pipeline for curating microarray data, **(C)** pipeline for pre-processing the microarray data, **(D)** methods used for meta-analysis of raw expression microarray data.

We conducted a meta-analysis using eight publicly available microarray expression datasets (Table 1) from varying tissues and microarray platforms on AD. We developed a thorough computational pipeline (Figure 1A) that involved curating and downloading raw microarray expression data, pre-processing the raw expression data and conducting a linear model analysis of the gene expression profiles. Statistically different genes based on disease state were identified following analysis of variance (ANOVA) on the linear model which compared gene expression changes due to disease state, sex, age, and tissue. These genes were further analyzed using a Tukey Honest Significant Difference (TukeyHSD) test to determine their biological significance (Tukey, 1949). In addition to the *p*-values, we also obtained the mean differences between binary comparisons of groups (also generated by the TukeyHSD), as a measure of biological effect size. We examined the TukeyHSD results by filtering by each factor, and identified up and down regulated genes. We then selected genes that showed statistically significant pairwise interactions between disease status and sex, age and tissue. Using these genes, we used R packages ReactomePA (Yu and He, 2016) and clusterProfiler (Yu et al., 2012) to conduct gene enrichment and pathway analyses of the differentially expressed genes (DEG). We used BINGO in Cytoscape v.3.7.0 for gene ontology (GO) analysis on each gene set for each factor (Shannon et al., 2003; Maere et al., 2005).

**Table 1:** Curated microarray datasets and the study description.

**Database**	**Accession number**	**Controls**	**AD**	**Possible AD**	**Platform**	**Citation**
GEO	GSE84422	242	362	449	Affymetrix Human Genome U133A, B and Plus 2.0	Wang et al., 2016
GEO	GSE28146	8	22	–	Affymetrix Human Genome Plus 2.0	Blalock et al., 2011
GEO	GSE48350	173	80	–	Affymetrix Human Genome Plus 2.0	Berchtold et al., 2008
GEO	GSE5281	74	85	–	Affymetrix Human Genome Plus 2.0	Liang et al., 2007
GEO	GSE63060	104	142	–	Illumina HumanHT-12 V3.0 expression beadchip	Sood et al., 2015
GEO	GSE63061	134	139	–	Illumina HumanHT-12 V4.0 expression beadchip	Sood et al., 2015
GEO	GSE29378	32	31	–	Illumina HumanHT-12 V3.0 expression beadchip	Miller et al., 2013
Array Express	E-MEXP-2280	5	7	–	Affymetrix Human Genome Plus 2.0	Bronner et al., 2009

### 2.1. Microarray Data Curation

We curated microarray expression data from two data repositories: National Center for Biotechnology Information (NCBI) Gene Expression Omnibus (GEO) (Edgar et al., 2002) and Array Express (Brazma et al., 2003) (Figure 1B). We searched these repositories by using entrez programming utilities in Mathematica (Mias, 2018b; Wolfram Research, Inc., 2017). In this search, we used the following keywords: *Homo sapiens*, Alzheimer's Disease and expression profiling by array (Figure 1B). This search resulted in 105 datasets from GEO and 8 from Array Express. We further filtered the search results by excluding data from cell lines, selecting for expression data from Illumina and Affymetrix microarray platforms, and focusing on datasets that provided the ages and sex of their samples (Figure 1B). After filtering through the databases, we found seven datasets from GEO (GSE84422, GSE28146, GSE48350, GSE5281, GSE63060, GSE63061, GSE29378) and one dataset from Array Express (E-MEXP-2280) to conduct our meta-analysis of expression profiling to assess differences in gene expression due to disease state, sex, age, and tissue (Table 1). The majority of samples from AD subjects were collected post-mortem, from a variety of brain banks, while the subjects from GSE63060 and GSE63061 voluntarily gave blood samples (Table S1). The criteria and guidelines followed for diagnosis and sampling varied across datasets (Table S1). Additionally, we downloaded the raw expression data from each dataset, and created a demographics file per study, which included characteristics about the samples (Table 2). Our demographics file included information about the subjects that was reported in all datasets. For example, some studies reported the type of AD diagnosis for their respective subjects, as well as the Braak stage and APOE genotype, whereas others did not (Table S1). Therefore, to ensure uniform annotation of the subjects, we re-annotated subject information provided from the databases: For GSE28146, we grouped the sub-types of AD, incipient, moderate and severe, as AD because we did not have such classification information for our other AD samples. We changed all the GSE29378 tissue types to hippocampus, relabeled the “probable AD” disease state to “possible AD” in GSE84422, only used AD and control subjects from the E-MEXP-2280 and GSM238944 with an age of >90 (not a definite age) was removed from GSE5281. We should note also that the 1,053 samples from the GSE84422 dataset included different tissues from the same subjects, which were treated independently—a paired-design was not incorporated in our downstream analysis.

**Table 2:** Patient characteristics for curated datasets.

**Accession number**	**Sex (M/F)**	**Age range**
GSE84422	302M/166F	60–103
GSE28146	12M/18F	65–101
GSE48350	124M/129F	20–99
GSE5281	102M/56F	63–102
GSE63060	88M/158F	52–88
GSE63061	107M/166F	59–95
GSE29378	38M/25F	61–90
E-MEXP-2280	7M/5F	68–82

### 2.2. Pre-processing and Data Normalization

We downloaded the raw expression data from the data repositories in Mathematica (Wolfram Research, Inc., 2017) and pre-processed each file in R (R Core Team, 2018) using the appropriate R packages based on the microarray platform. The affy package was used to pre-process all the .CEL data files from Affymetrix (Gautier et al., 2004), and the limma package for Illumina summary data files (Ritchie et al., 2015). We performed background correction, normalization and annotated and summarized all probes (Figure 1C). For the Affymetrix expression data files, we used the expresso function with the following parameters: robust multi-array analysis (RMA) for background correction, perfect-match (PM) adjustment to correct the perfect match probes, and ‘avdiff’ for the summary method to compute expression values (Gautier et al., 2004). We also used the avereps function from limma to summarize probes and remove replicates (Ritchie et al., 2015). For the Illumina expression data, we corrected the background using the NormExp Background Correction (nec) function from the limma package for datasets where the detection *p*-values were reported, we annotated and used the aggregate function from the stats package in base R to summarize probes (Ritchie et al., 2015; R Core Team, 2018). We merged all 8 datasets into one large matrix file via common gene symbols. After merging the datasets, we performed a BoxCox power transformation (Sakia, 1992) using the ApplyBoxCoxTransform function and data standardization using the StandardizeExtended function from the MathIOmica package (Mias et al., 2016; Mias, 2018b) (Figure 1C and also see ST2 of online Supplementary Datasheet).

### 2.3. Visualizing Variation Due to Batch Effects

Merging expression data from different studies, array platforms and tissues can introduce confounding factors and manipulate interpretation of results. To address this, and assess whether batch effects were evident and could be accounted for, we used the ComBat function in the sva package in R (Johnson et al., 2007; Nygaard et al., 2016) to adjust data for known batch effects. In this study, the batch effect was the study (i.e. different experiments/research groups), and we also found that there was a one-to-one correspondence between study and platform. Using expression data from prior to and post ComBat corrections, we used principal component analysis (PCA) plots to visualize the variability in the data and the effectiveness of possible batch effect removal (Irizarry and Love, 2015).

### 2.4. Analysis of Variance

We modeled the merged expression data (see model breakdown below) prior to running ANOVA (using the anova and aov functions from the stats package in base R) to analyze differences among the different study factors (Figure 1D) (Pavlidis, 2003). We defined age group, sex, disease state, study and tissue as factors.

(1) $$\begin{array}{r} x\sim \;\underset{i}{\sum }x_{i}+\underset{i,j;j>i}{\sum }x_{i}:x_{j} \end{array}$$

where *x*_*i*_ ∈ {age group, sex, tissue, disease status} and the factors have the following levels:

- disease status = {control, possible AD, AD}
- sex = {male, female}
- age group = {under 60, 60–65, 65–70, 70–75, 75–80, 80–85, 85–90, 90–95, over 95}
- tissue = {amygdala, anterior cingulate, blood, caudate nucleus, dorsolateral prefrontal cortex, entorhinal cortex, frontal pole, hippocampus, inferior frontal gyrus, inferior temporal gyrus, medial temporal lobe, middle temporal gyrus, nucleus accumbens, occipital visual cortex, parahippocampal gyrus, posterior cingulate cortex, precentral gyrus, prefrontal cortex, primary visual cortex, putamen, superior frontal gyrus, superior parietal lobule, superior temporal gyrus, temporal pole}
- study = {GSE84422, GSE28146, GSE48350, GSE5281, GSE63060, GSE63061, GSE29378, E-MEXP-2280}

The *p*-values following the ANOVA were adjusted using Bonferroni correction for multiple hypothesis testing (Pavlidis, 2003). Genes with *p*-values < 0.05 were considered statistically significant. We found statistically significant disease genes by filtering on the disease status for *p* < 0.05. Additionally, we used the enrichKEGG function in the clusterprofiler package in R for Kyoto Encyclopedia of Genes and Genomes (KEGG) enrichment analysis on these genes (Kanehisa and Goto, 2000; Yu et al., 2012). We also performed Reactome pathway analysis with the enrichPathway function in the ReactomePA package in R (Yu and He, 2016). These packages adjust *p*-values using the Benjamini Hochberg method for False Discovery Rate (FDR) control. Enriched pathways with adjusted *p* < 0.05 were considered statistically significant (Yu et al., 2012; Yu and He, 2016) (see ST5 and ST6 of online Supplementary Datasheet).

### 2.5. Identifying Up and Down Regulated Genes by Factor

To identify which of the 3,735 genes that show biologically significant differences, we conducted a TukeyHSD (using the TukeyHSD function from the stats package in base R) to determine statistically significant up and down-regulated genes using the difference in the means of pairwise comparisons between the levels within each factor (Tukey, 1949; Mias, 2018a). We carried out TukeyHSD testing on the statistically significant disease genes we obtained from the ANOVA. To account for multiple hypothesis testing in the TukeyHSD results, we used <0.00013 (0.05/number of genes ran through TukeyHSD) as a Bonferroni adjusted cutoff for statistical significance.

We selected the TukeyHSD results from the disease status factor, and focused on the “Control-AD” pairwise comparison to assess statistically significant gene expression differences. To assess biological effect, and select an appropriate fold-change-like cutoff (as our results had already been transformed using a Box-Cox transformation), we calculated the quantiles based on the TukeyHSD difference of mean difference values (Table S2). We used a two-tailed 10 and 90% quantile to identify significantly up and down regulated genes (Table S2).

The DEG by disease status factor were subsequently used to determine whether or not there was a sex, age, or tissue effect on them. For sex, we used the DEG to filter the TukeyHSD results for sex factor differences, identified statistically significant sex-relevant genes based on *p*-value cutoff, and the computed 10 and 90% quantiles based on the difference of means between male and female groups. We repeated the above steps for age group, but focused only on the binary comparisons where all age groups were compared to the <60 age group, which was used as a baseline (i.e. computed the mean gene expression differences per group comparison, *i*- <60, where *i* stands for any age group). This was carried out to enable us to compare the progression with age, relative to a common reference across all age groups. As for tissue, we carried out the same steps as above to determined DEG based on comparisons both a hippocampus-based baseline, as well a blood-based baseline.

Following the identification of the DEG by disease status and sex, we visualized the raw expression data for these genes in heatmaps. In addition to this, we generated heatmaps using the difference of means values (TukeyHSD) for the identified DEG by age group (<60 baseline) and tissue (hippocampus and blood as baseline).

To further investigate the significance of pairwise interactions with disease status and the factors sex, age and tissue, we used the identified statistically significant (*p* < 0.00013, two-tailed 10 and 90% quantile) genes from our *post-hoc* analysis for each factor, and filtered our ANOVA results for statistically significant interactions (Bonferroni corrected *p* < 0.05, see also ST4 of online Supplementary Datasheet).

### 2.6. Gene Ontology and Reactome Pathway Analysis

For the disease and sex DEG sets, we used the R package ReactomePA to find enriched pathways (Yu and He, 2016). We also built networks to determine if genes overlapped across pathways. Additionally, we used BINGO in Cytoscape for GO analysis to determine the biological processes the genes were enriched in Maere et al. (2005). Results were considered statistically significant based on Benjamini-Hochberg adjusted *p*-value < 0.05.

## 3. Results

With our data selection criteria outlined in Figure 1B we identified 8 datasets from GEO and Array Express to conduct our meta-analysis to assess differences in gene expression due to disease state, sex, age, and tissue (Table 1). We merged the processed expression data by common gene names, which gave us a total of 2,088 samples and 16,257 genes. The 2,088 samples consisted of 771 healthy controls, 868 AD subjects, 449 subjects reported as possibly having AD, 1308 females, and 780 males.

### 3.1. ComBat Batch Effect Visualization

**Figure 2:**
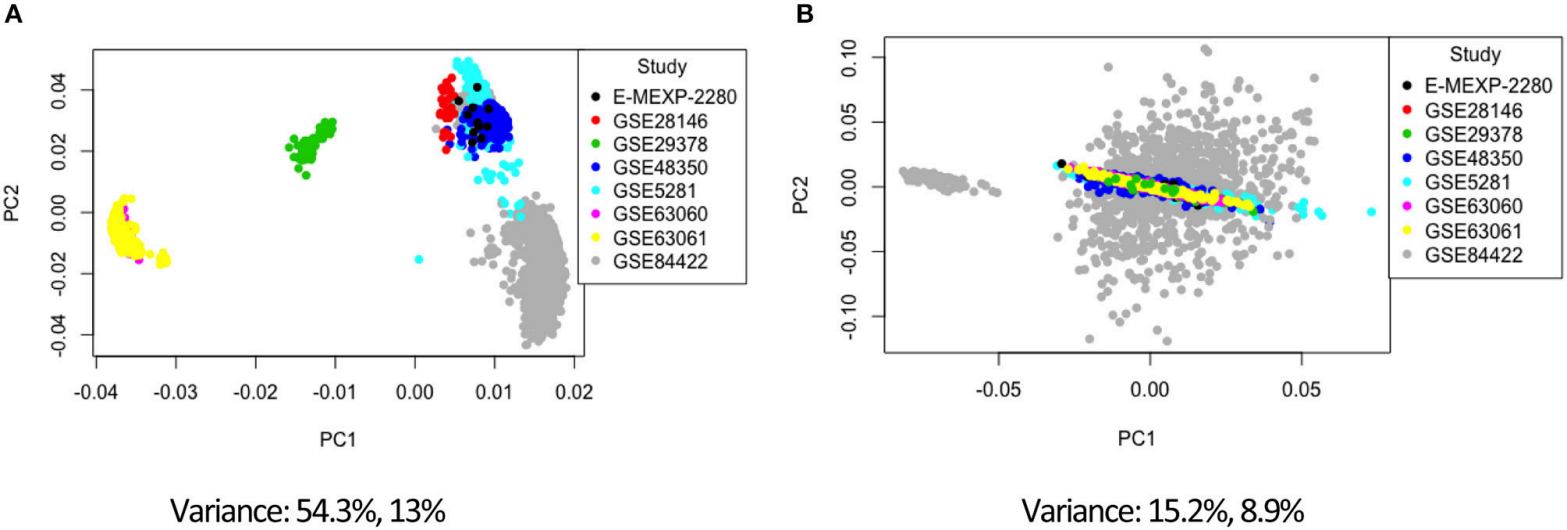
Principal component analysis of the study factor before **(A)** and after **(B)** batch correction with ComBat.

**Figure 3:**
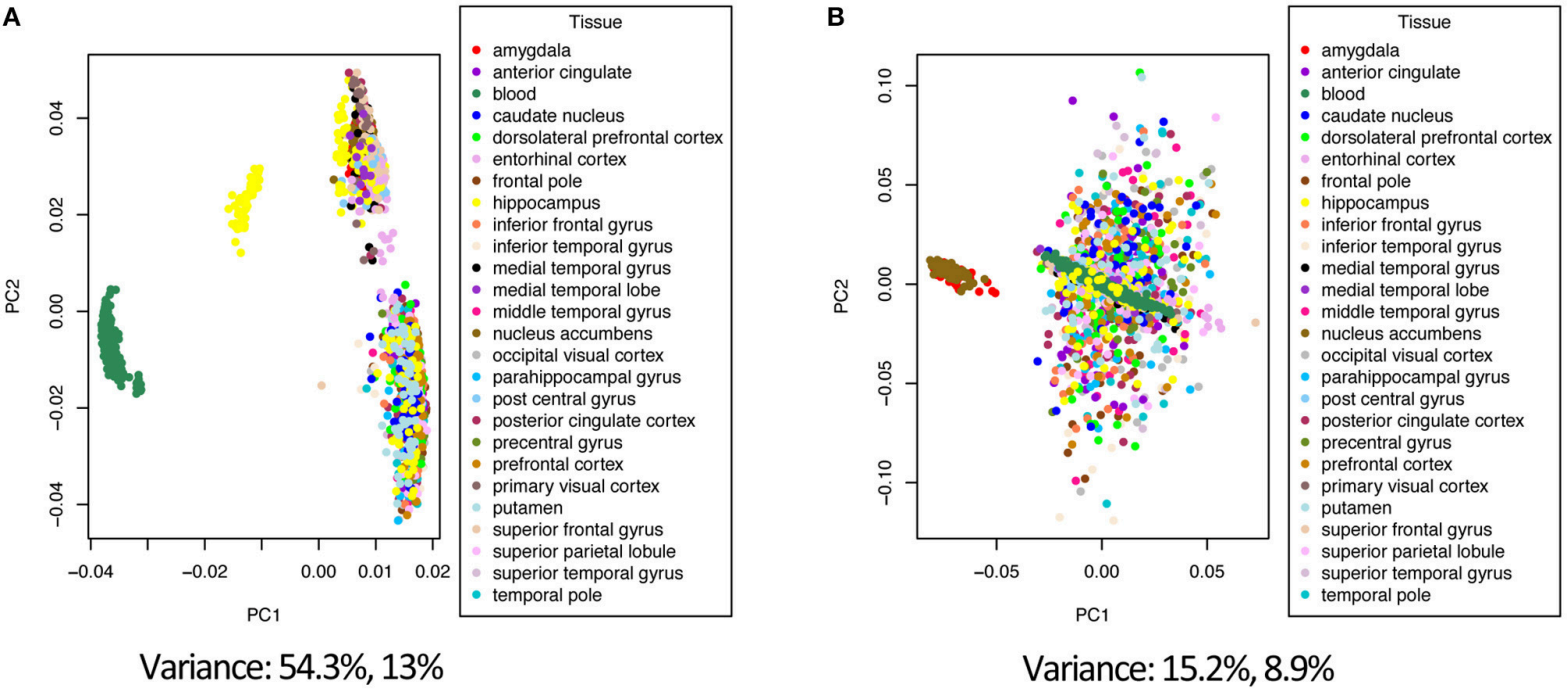
Principal component analysis of the tissue factor before **(A)** and after **(B)** batch correction with ComBat.

Combining data from different platforms, tissues and different laboratories introduces batch effects. Batch effects are sources of non-biological variations that can affect conclusions. We used the ComBat algorithm in R which works by adjusting the data based on a known batch effect. For our analysis we classified the study variable as our batch (the study and type of platform are directly related). We used PCA to visualize variation in the merged expression data before and after ComBat (Figures 2, 3; Figures S1–S3). In Figure 2 before correcting for batch effects, the datasets separate into four main clusters with a variance of 54.3% in PC1 and 13% in PC2. Following ComBat, those main clusters appear to be removed, with an overall reduction in variation for both principal components. We also looked at how the data separated by factor. In Figure 2B, there are two clear groups and this separation is accounted for when we look at the separation in the data by tissue (Figure 3). In Figure 3, before correction the four groups observed in Figure 2 are still evident. Following ComBat, the tissues: amygdala and nucleus accumbens cluster together in one group while all other tissues are in another. Batch effect correction with ComBat was solely used for visualizing how the expression data separates before and after ComBat correction—i.e., the batch corrected expression data were not used in the downstream analysis. We instead used a linear model to account for confounding study effects. Visualizing and understanding the variation within the expression data following the merge confirmed the need to include the study as a factor in the linear model analysis.

### 3.2. Analysis of Variance on Gene Expression by Disease State

**Figure 4:**
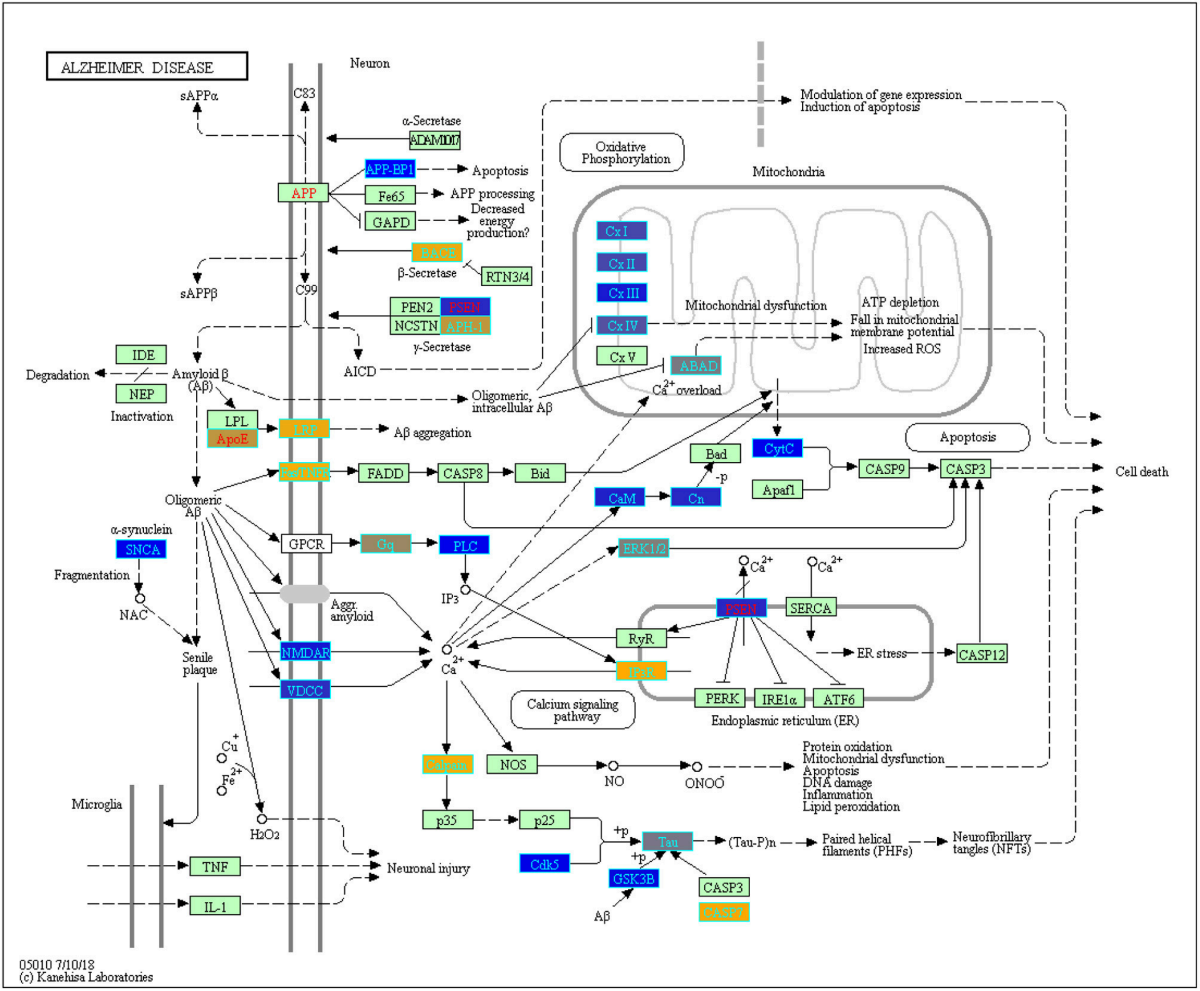
Enriched genes from the ANOVA statistically significant disease status gene list (*p*-value < 0.05) found in the KEGG Alzheimer's disease pathway (hsa05010) [Kanehisa and Goto, 2000; Kanehisa et al., 2016, 2017]. The yellow shading represents up-regulated and the blue shading represents down-regulated in AD samples. These genes were not yet filtered for biological significance.

Using ANOVA we assessed the variance in gene expression across the different factors in our linear model by including the following factors and their pairwise interactions: age group, study, tissue, sex and disease state (Pavlidis, 2003). Statistically significant gene expression differences were determined using a Bonferroni (Bland and Altman, 1995 adjusted *p* < 0.05) (Pavlidis, 2003; Mias, 2018a). With our focus on differences by disease status, we filtered genes based on the ANOVA adjusted *p*-values for the disease factor. Selecting for statistical significance by disease status we found 3,735 genes (see ST4 of online Supplementary Datasheet). We conducted GO and pathway analysis on these genes. The KEGG pathway analysis results are displayed in Table 3 (see ST5 of online Supplementary Datasheet for full table). The analysis showed that the genes are involved in Reactome pathways, such as the Mitochondrial Translation Initiation (55 gene hits), Signaling by the B Cell Receptor (61 gene hits), Activation of NF-kappaβ in B cells (40 gene hits), Transmission across Chemical Synapses (83 gene hits) and Neuronal System (119 gene hits) (see ST6 of online Supplementary Datasheet). The KEGG pathways that were enriched for this gene set included neurodegenerative disease pathways, such as Alzheimer's (31 gene hits), Huntington's (76 gene hits) and Parkinson's (53 gene hits) (Table 3) Pathways. We also had genes enriched in synaptic pathways including Synaptic vesicle cycle (30 gene hits), Dopaminergic synapse (48 gene hits) and GABAergic synapse (34 gene hits) (Table 3). In addition to synapses and neurodegeneration, the long term potentiation (23 gene hits) pathway was associated with these genes (see ST5 of online Supplementary Datasheet for full KEGG pathway analysis results). To further explore the enriched genes in the KEGG AD pathway, we used the TukeyHSD results to determine whether genes were up- or down-regulated (see ST7 of online Supplementary Datasheet). To further assess the 73 gene hits identified in the enriched AD pathway we computed their mean differences between AD and control subjects, and used MathIOmica (Mias et al., 2016) tools to highlight them in the AD pathway (Figure 4) (Kanehisa and Goto, 2000; Kanehisa et al., 2016, 2017; Mias, 2018b) (see ST7 of online Supplementary Datasheet for full table with difference of means). For instance, the APOE and LRP gene were both found to be up-regulated in AD subjects compared to healthy controls, and in the KEGG AD pathway these genes are involved in Aβ aggregation (Figure 4).

**Table 3:** Top 25 KEGG Pathways using differentially expressed genes.

**ID**	**Description**	***p*-value**	***p*-adjusted value**	**# of hits**
hsa03050	Proteasome	1.55E-11	4.78E-09	31
hsa04723	Retrograde endocannabinoid signaling	3.46E-10	4.78E-08	66
hsa05010	Alzheimer's disease	4.64E-10	4.78E-08	73
hsa00190	Oxidative phosphorylation	3.85E-09	2.98E-07	59
hsa05016	Huntington's disease	1.60E-08	9.90E-07	76
hsa04714	Thermogenesis	2.54E-08	1.31E-06	86
hsa04932	Non-alcoholic fatty liver disease (NAFLD)	2.98E-06	1.32E-04	57
hsa04721	Synaptic vesicle cycle	4.57E-06	1.77E-04	30
hsa05012	Parkinson's disease	1.51E-05	5.18E-04	53
hsa04728	Dopaminergic synapse	6.48E-05	0.002003299	48
hsa04724	Glutamatergic synapse	1.58E-04	0.004085366	42
hsa05169	Epstein-Barr virus infection	1.59E-04	0.004085366	66
hsa04720	Long-term potentiation	1.73E-04	0.004119762	28
hsa04727	GABAergic synapse	2.31E-04	0.00506623	34
hsa01200	Carbon metabolism	2.46E-04	0.00506623	42
hsa01521	EGFR tyrosine kinase inhibitor resistance	3.12E-04	0.006031187	31
hsa04725	Cholinergic synapse	4.73E-04	0.008596289	40
hsa00270	Cysteine and methionine metabolism	5.56E-04	0.009547497	20
hsa04911	Insulin secretion	5.99E-04	0.009738112	32
hsa04713	Circadian entrainment	6.78E-04	0.01048273	35
hsa05033	Nicotine addiction	8.70E-04	0.012730978	18
hsa00650	Butanoate metabolism	9.06E-04	0.012730978	14
hsa03010	Ribosome	0.0010736	0.014423588	50
hsa04510	Focal adhesion	0.001159439	0.014927779	62
hsa04390	Hippo signaling pathway	0.001260878	0.015584456	50

### 3.3. Up and Down- Regulated Gene Expression in AD and Sex Specific Differences

**Figure 5:**
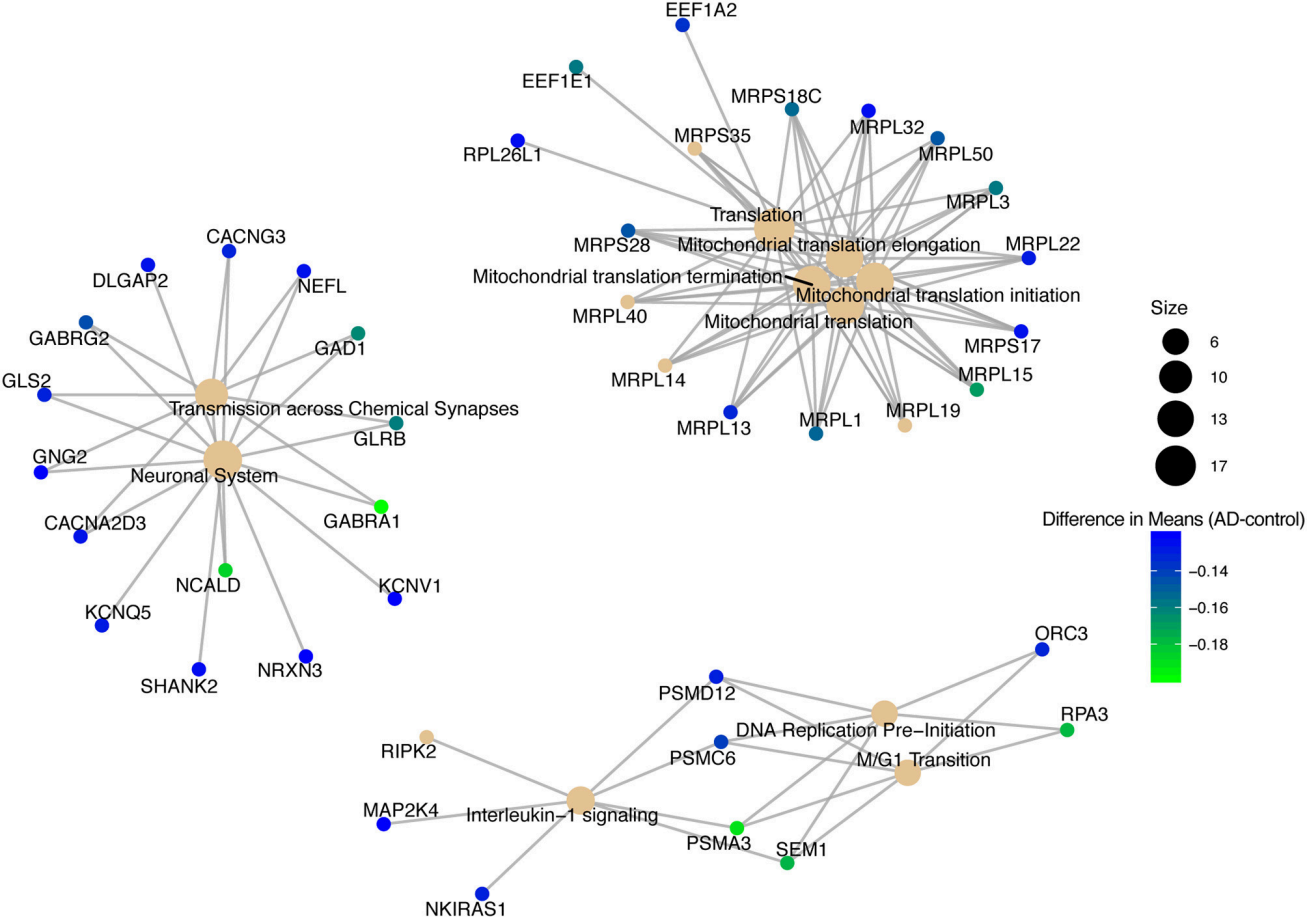
Pathway-gene network of top 10 enriched Reactome pathways from down-regulated genes in Alzheimer's disease patients.

**Figure 6:**
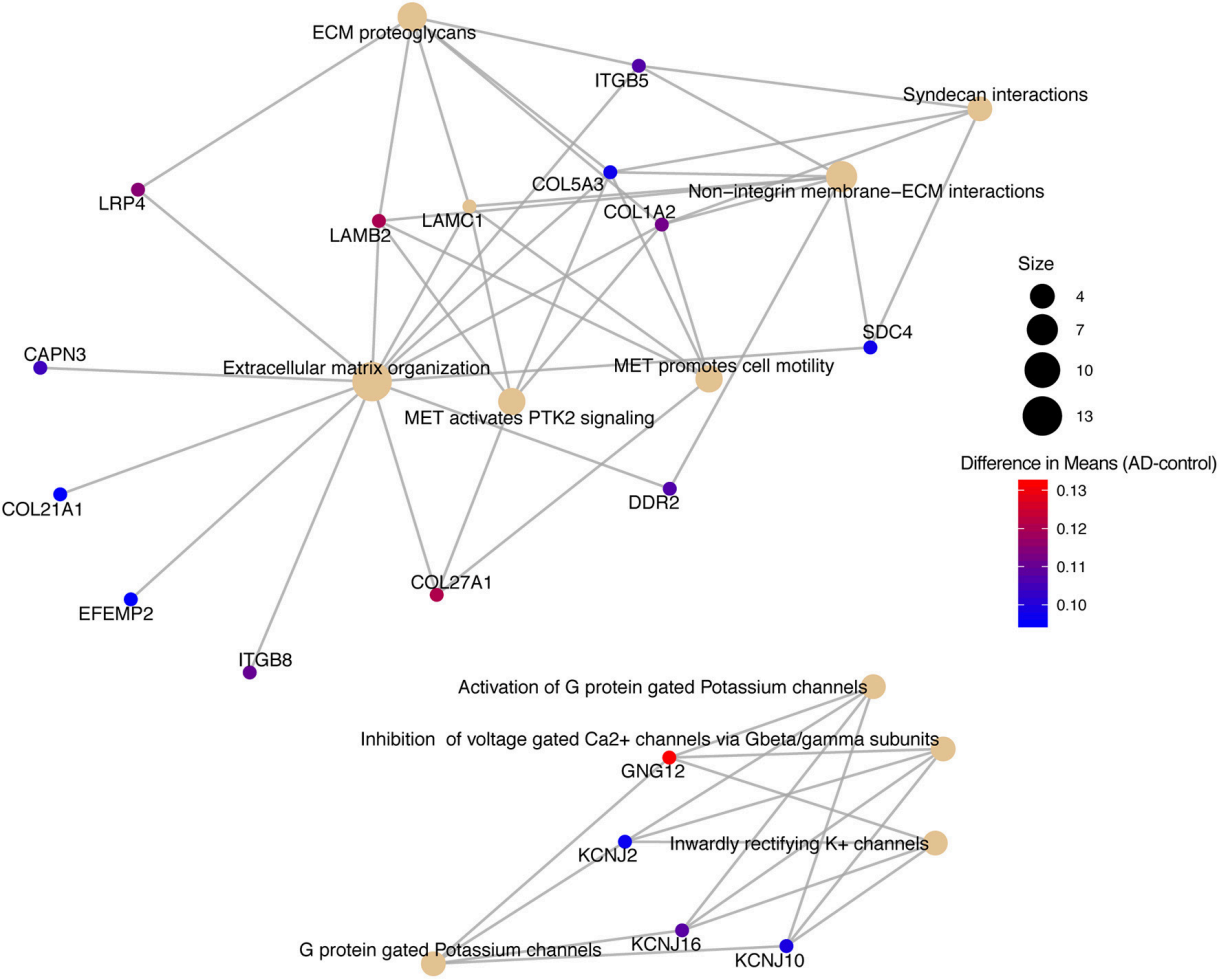
Pathway-gene network of top 10 enriched Reactome pathways from up-regulated genes in Alzheimer's disease patients.

We conducted a *post-hoc* analysis (TukeyHSD) on the 3,735 statistically significant disease genes to identify factorial differences and explore up- and down- regulation of genes. We were particularly interested in the control compared to AD gene expression differences, and how these could be further sub-categorized to explore effects by sex, age and tissue. We used a Bonferroni adjusted *p*-value cut off for significance (<0.000013) and the 10% two-tailed quantile to determine significantly up and down regulated genes (Table S2). In the Control-AD TukeyHSD comparisons, we found 352 statistically significant genes that we classified as up-regulated (176 DEG) and down-regulated (176 DEG) in AD subjects (or correspondingly up or down- regulated in controls) if their mean differences were ≤ −0.0945 and ≥ 0.1196, respectively (Table S2, see also ST8 of online Supplementary Datasheet). The top 25 up- and down- regulated genes sorted by the TukeyHSD adjusted *p*-values are outlined in Table 4 (Figure S4 and see ST8 of online Supplementary Datasheet). After performing gene enrichment and pathway analysis with the ReactomePA R package (Yu and He, 2016) on the 352 genes we built pathway-gene networks for the statistically significant Reactome pathways (Benjamini-Hochberg adjusted *p* < 0.05) (see ST13 and ST14 of online Supplementary Datasheet). Some of the top 10 enriched Reactome pathways from DEG down-regulated in AD include: Mitochondrial translation elongation, Mitochondrial translation, Transmission across chemical synapses, neuronal system (Figure 5 and Figure S5). The network in Figure 5 illustrates that some genes overlap across pathways—the difference of means from the TukeyHSD results of these genes are indicated by the color scale. The up-regulated genes in AD were enriched in pathways, such as Extracellular matrix (ECM) organization and ECM proteoglycans, Non-integrin membrane-ECM interactions and potassium channel activation (Figure 6 and Figure S6). Additionally, we used BINGO for GO analysis on the 352 disease DEG to determine the biological processes they are involved in Figure S7. Some examples of significant terms: Cell signaling development, nervous system development, neuron differentiation, cell proliferation, response to chemical stimulus, cell communication and brain and nervous system development (Figure S7).

**Table 4:** Top 25 up- and down-regulated genes in Alzheimer's disease compared to healthy controls.

**Up-regulated**		**Down-regulated**	
**Gene**	**Difference of means**	**Gene**	**Difference of means**
ITPKB	0.1709575	RPA3	−0.1781622
ARHGEF40	0.1574220	NME1	−0.1755078
CXCR4	0.1907433	LSM3	−0.1527917
PRELP	0.1319160	MRPL3	−0.1577078
SLC7A2	0.1568425	PTRH2	−0.1205413
AHNAK	0.1304494	RGS7	−0.1778522
NOTCH1	0.1014441	GLRX	−0.1622333
GFAP	0.1198343	RPH3A	−0.2168597
HVCN1	0.1151989	BEX4	−0.1416335
LDLRAD3	0.1627433	COX7B	−0.1726039
KANK1	0.0992824	NRN1	−0.1634702
HIPK2	0.1255059	PPEF1	−0.1430548
SLC6A12	0.1485253	PCSK1	−0.3127961
KLF4	0.1870071	ENY2	−0.1496523
ABCA1	0.1386346	CD200	−0.1537059
DDR2	0.1069751	NRXN3	−0.1203814
KLF2	0.1070143	GTF2B	−0.1508171
GNG12	0.1318200	MRPS18C	−0.1535766
POU3F2	0.1022426	NCALD	−0.1858802
AEBP1	0.1498719	C11orf1	−0.1448555
IQCA1	0.1134073	DCTN6	−0.1222108
ERBIN	0.1309312	SEM1	−0.1765024
LOC202181	0.1184466	APOO	−0.1384320
LPP	0.1072798	CCNH	−0.1394853
NOTCH2	0.1213843	RAD51C	−0.1280948

Of the 352 DEG in the above disease analysis, 46 genes were differentially expressed by sex: 23 down- and 23 up-regulated in males compared to females (Table S2) based on mean differences (≤ −0.0864 and ≥ 0.2502 respectively) (Table S1). We used the ReactomePA package to build a network of enriched genes and pathways with sex differences (Figure S8) (Yu and He, 2016). We found 6 pathways that were enriched with the up-regulated gene list in males: Neuronal System, Transmission across chemical synapses, neurotransmitter receptors, and post-synaptic signal transmission, and GABA A receptor activation (Figure S8 and also see ST9 of online Supplementary Datasheet). Of these 46 genes that were differentially expressed by sex (Figure S9), we further filtered the ANOVA results to identify which of these genes showed statistically significant interactions with disease (sex:disease, Bonferroni corrected *p* < 0.05). We found one gene, chemokine receptor type 4 (CXCR4), to have a statistically significant pairwise interaction between disease status and sex (see ST4 of online Supplementary Datasheet).

### 3.4. Aging and Tissue Differences in AD Gene Expression

**Figure 7:**
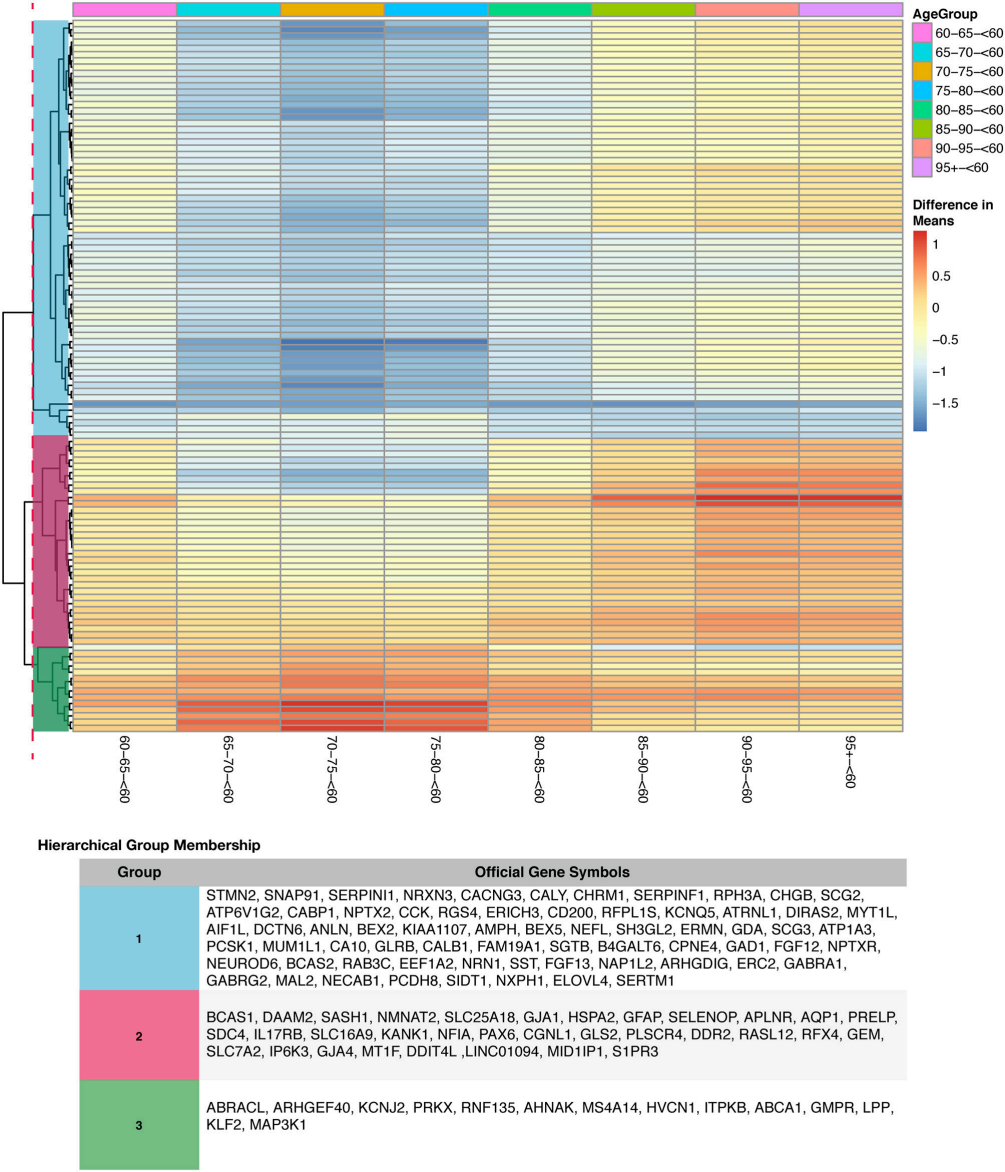
Heatmap with gene clustering to visualize age group effect (difference in means) on the differentially expressed disease (control-AD) gene list that have agegroup:disease status interaction.

To determine if age or tissue had an effect on the DEG by disease status, we filtered the 352 DEG in disease results discussed above for age group and tissue comparisons. For age effects, we used our TukeyHSD results that compared age groups to <60 (served as the baseline). This allowed us to explore if genes associated with AD change with age by using a common reference group. We used the 352 DEG genes from disease status TukeyHSD results to find sizable age effects in this gene set by selecting for statistical significance and using the two-tailed 10% quantile filter (≤ −1.0477827 and ≥ 0.330869) to find significant DEG per age-group pair comparison (Table S2). We found 396 significant comparisons of age differences in 141 genes (see ST10 of online Supplementary Datasheet). The 141 genes were plotted across all age comparisons where <60 was the baseline to visualize expression changes and how the genes clustered (Figure S10), indicative of distinct differences in expression profiles due to aging. There is a cluster of genes down-regulated in older age groups, specifically ages 65–80 compared to those <60. There also appears to be an overall trend of genes associated with disease being up-regulated compared to <60. Of the 141 DEG by age group (Figure S10), we found 114 DEG that had a statistically significant interaction (Bonferroni corrected *p* < 0.05) between disease status and age (Figure 7). Changes in expression across each age group comparison (<60 baseline) in the interacting genes were visualized, and the genes clustered into 3 clear groups based on similarities in expression patterns (Figure S10).

**Figure 8:**
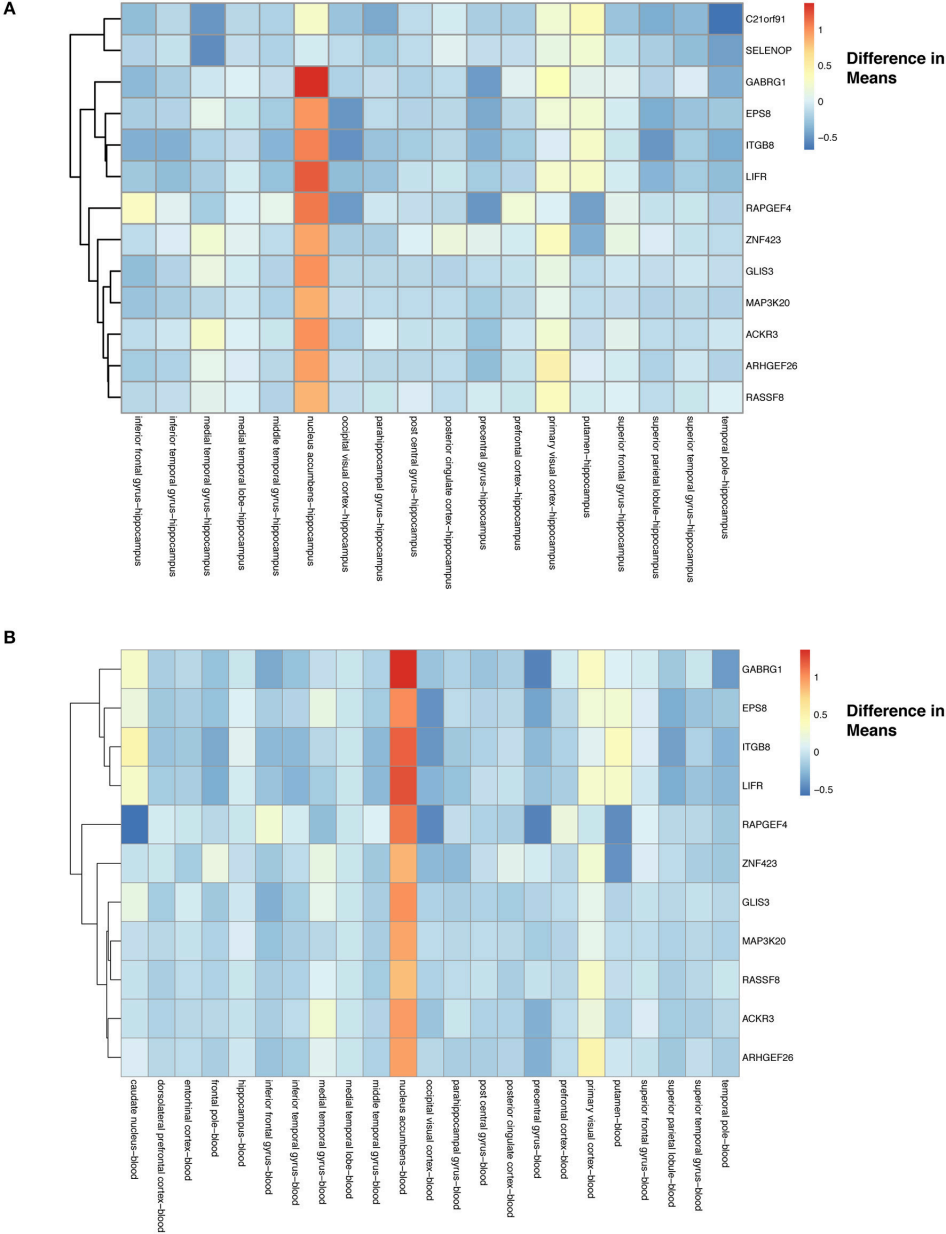
Heatmap with gene clustering to visualize tissue effect (difference in means) on the differentially expressed disease (control-AD) gene list that have tissue:disease status interaction. **(A)** Difference in means using hippocampus as the baseline. **(B)** Difference in means using blood as the baseline.

For tissue effects, we used hippocampus as our baseline due to it being a known target of AD. In addition to filtering for significance, we used again a two-tailed 10% quantile filter ≤ −0.6359497 and ≥ 0.7932871 from the tissue-specific means differences between tissue types (Table S2). We found 167 comparisons with tissue differences (see ST11 of online Supplementary Datasheet) from 125 genes. Our heatmap of these genes show that differences do exist across tissues when compared to hippocampus (Figure S11). For example, nucleus accumbens has higher expression of genes compared to the hippocampus, and putamen has genes that are down-regulated compared to hippocampus (Figure S11). The majority of the expression differences appear to be found in nucleus accumbens and putamen (Figure S11, see also ST11 of online Supplementary Datasheet). From these 125 tissue specific (hippocampus) genes, we found 13 to have a statistically significant (Bonferroni corrected *p* < 0.05) interaction between disease and tissue (Figure 8A).

We also assessed how gene expression changes in a given tissue compared to blood (10 %quantile filter: ≤ −0.6359497 and ≥ 0.7932871) (Table S2), identifying 152 significant tissue comparisons in 115 genes (see ST12 of online Supplementary Datasheet). These 115 gene expression profiles across tissues are visualized using the differences of means in Figure S11. We again noticed similar trends in the blood comparisons as had in the hippocampus comparisons, with nucleus accumbens showing higher gene expression and putamen lowered expression compared to blood (Figure S12). Finally, we found that 11 of these genes had a statistically significant (Bonferroni corrected *p*-value < 0.05) interaction between disease and tissue (Figure 8B).

## 4. Discussion

As debilitating as Alzheimer's disease (AD) is, there is still no cure available, and diagnosis is not confidently confirmed until death. There are ongoing research efforts to find biomarkers and gene targets for early detection and intervention in AD. In our study, we investigated changes at the transcript level by conducting a meta-analysis to analyze eight microarray expression datasets for temporal changes in gene expression due to disease status. In addition to this, we determined if sex, age, or tissue type had an effect on gene expression changes in Alzheimer's associated disease genes. We pre-processed the eight datasets by background correction, data normalization, and probe annotation. Following this, the datasets were merged into a single dataset (by common gene name) for the meta-analysis. This is the first meta-analysis to explore over 20 different tissues and use a linear model to identify linear and binary effects on gene expression. Our linear model also adjusted batch effects by modeling for the study effect and included age in the model as a linear time series. Modeling with the study factor to account for batch effects was shown to be necessary after exploratory visualization of the expression data before and after combat batch effect correction using principal component analysis to remove variation within the data that was introduced due to different studies (Figures 2, 3).

### 4.1. Significant Gene Expression Differences Due to Disease Status and Biological Significance

We first identified statistically significant disease genes (*p* < 0.05; factor: disease status) from ANOVA (see ST4 of online Supplementary Datasheet), and these genes included: APOE, PSEN2, APOD, TREM2, CLU which all have been previously associated with AD. APOE and APOD are members of the apolipoprotein family that transport and metabolize lipids in the central nervous system and play a role in healthy brain function (Elliott et al., 2010). APOE is a strong, well documented, genetic risk factor for AD, and polymorphisms in APOE have been shown to affect age of AD onset (Masters et al., 2015). APOD's mechanism is still not completely understood (Elliott et al., 2010), PSEN2 encodes presenilin-2, an enzyme that cleaves APP, regulates production of Aβ, and mutations are associated with early onset (Masters et al., 2015). Mutations in CLU lead to lower white matter and increases AD risk (Braskie et al., 2011; Masters et al., 2015) and TREM2 was identified by a genome-wide association study (GWAS) as a disease variant and risk factor for AD (Masters et al., 2015). Our enrichment results of the 3,735 genes (from ANOVA) were interesting due to them having already been associated with AD in the literature (Table 3 and also see ST5, ST6 of online Supplementary Datasheet). For instance, mitochondrial dysfunction has been previously associated with AD and characterized to cause Aβ deposition, higher production of reactive oxygen species and lowered ATP production (Moreira et al., 2010; Onyango et al., 2016; Swerdlow, 2018). Researchers have also suggested that the immune system plays a role in AD (Heppner et al., 2015; Van Eldik et al., 2016). As for adaptive immune cells, their role in AD is still not clear, however, adaptive immune cells have been shown to reduce AD pathology (Marsh et al., 2016). The loss of B cell production can exacerbate the disease (Marsh et al., 2016). Neurodenegenerative diseases have also been described as having genes that overlap (Wang et al., 2017; Moradifard et al., 2018). Neurodegeneration is closely related to synaptic dysfunction and long term potentiation becomes impaired with age and synaptic dysfunction (Prieto et al., 2017). These results suggest that our meta-analysis is producing disease-related results (Table 3 and also see ST5, ST6 of online Supplementary Datasheet).

We also identified the KEGG AD pathway as one of our enriched pathways based on the 3,735 statistically significant disease genes. To explore how these genes are regulated in the AD pathway, we used the difference of means (using the TukeyHSD) to create (Figure 4) which highlights 73 of the 3,735 genes from our ANOVA analysis and their role in the KEGG AD pathway (see ST7 of online Supplementary Datasheet). NAE1, also known as amyloid precursor protein-binding protein 1 (APP-BP1), was down-regulated in AD subjects and is involved in neuronal apoptosis (Figure 4). The literature indicates that APP-BP1 is necessary for cell cycle progression and activates the neddylation pathway that drives apoptosis (Chen et al., 2000, 2003, 2007, 2012; Laifenfeld et al., 2007; Zhang et al., 2015). Down-regulation of APP-BP1 has been associated with increased APP while over expression of APP-BP1 leads to APP degradation (Chen et al., 2000, 2003, 2007, 2012; Laifenfeld et al., 2007; Zhang et al., 2015). TNFRSF6 was up-regulated in AD subjects (Figure 4, and this gene produces the Fas antigen which plays a role in mediating apoptosis (Feuk et al., 2000).

The KEGG AD pathway also highlights genes from our analysis that are involved in APP processing and cleavage (Figure 4). Specifically, BACE, PSEN, and APH-1 are all involved in APP processing by coding for γ-secretase and β-secretase (Figure 4). BACE is a β-secretase, that we found to be up-regulated in AD subjects compared to controls (Figure 4). This finding also supports previous reports that BACE is over-expressed in AD brains, and plays a role in forming Aβ (Vassar, 2004; Das and Yan, 2017). APH-1A and PSEN2 are a part of the γ-secretase complex that finalizes cleavage and release of APP to produce Aβ (Serneels et al., 2005; De Strooper and Annaert, 2010; Jurisch-Yaksi et al., 2013). As shown in Figure 4, in AD subjects there was a high production of APH-1 while PSEN2 was down-regulated. This indicates that while in a complex, the two genes may function differently. For example, mutations in PSEN2 can lead to memory loss and loss of synaptic plasticity (Saura et al., 2004). A better understanding of the mechanistic behavior of the γ-secretase complex genes can aid in the potential development of targeted therapeutics for γ-secretase. Also in the AD pathway we found up-regulated expression of APOE and LRP1 in AD subjects compared to control subjects (Figure 4). These genes are both involved in Aβ aggregation. LRP1 a known receptor of APOE and promotes Aβ aggregation and migration across blood-brain barriers (O'Callaghan et al., 2014).

As discussed above, mitochondrial dysfunction is a key hallmark of AD. Genes from our meta-analysis that are in the AD pathway are involved in the respiratory electron chain transport complexes. For example, NDUFC2 (in CxI on Figure 4), SDHA (in CxII on Figure 4), and COX5B, COX6A1, COX6C (in CxIV) are all necessary for electron transport, but were down-regulated in AD (Figure 4). In Figure 4, complexes I-IV of the electron chain transport were all down-regulated in AD. Previous work observed lower expression of 70% of genes that code for subunits of the electron transport chain (Liang et al., 2007). Reduced mitochondrial translation and lowered mRNA levels for genes, such as cytochrome oxidase (COX), can lead to increased oxidative stress, irregular calcium levels and decreased oxidative phosphorylation (OXPHOS) (Chandrasekaran et al., 1994, 1997; Parker et al., 1994; Markesbery, 1997; Liang et al., 2007; Bi et al., 2018). Hence, changes due to mitochondrial dysfunction may affect the pathology of neurodegenerative diseases, such as AD.

We also found ITPR3, a gene involved in the calcium signaling pathway, was up-regulated in AD (Figure 4). ITPR3 is necessary for the release of Ca^2+^ from the endoplasmic reticulum (Berridge, 2016). Increased expression of this gene and calcium concentrations can cause memory loss and neuron cell death (Figure 4) (Berridge, 2016). Additionally, we found genes involved in tau phosphorylation to be up-regulated in AD (Figure 4). Calpain (CAPN1, CAPN2) which is activated by elevated levels of cytostolic calcium is up-regulated as well as CASP7 (Ferreira, 2012). Together these genes regulate tau phosphorylation and the formation of neurofibrillary tangles, which eventually leads to neuronal cell death (Figure 4).

In addition to enrichment in the AD pathway, our KEGG results on the 3,735 genes included enrichment in Parkinson's disease and Huntington's disease pathways. Because of this we investigated if the three neurodegenerative disease signaling pathways had any common genes in our gene list (Table 3). We determined that AD had 49 genes that overlapped with Huntington's and 47 with Parkinson's pathways respectively. We also found that GNAQ, GRIN1, and PLCB1 are in both Huntington's and AD but not in Parkinson's pathways, and SNCA is in both Parkinson's and AD but not Huntington's pathways. In filtering the statistically significant disease genes for biological effect size (*post-hoc* analysis), PSEN2, APOE, TREM, CLU, and other apolipoproteins did not make the cutoff (based on their difference in means between the compared AD/healthy groups).

Focusing on the 352 DEG that had a sizable biological effect, the down-regulated genes in AD connect with the pathology of the disease (Figure 5). Specifically, genes in the Mitochondrial translation pathway that were down-regulated in AD included MRPL15, MPRL13, and MRPL1, which are all mitochondrial ribosomal proteins necessary for protein synthesis (Pearce et al., 2013; Stelzer et al., 2016; Fabregat et al., 2017). These genes may also be related to down-regulation of the mitochondrial electron transport chain complexes (Bonilla et al., 1999) in the KEGG AD pathway (Figure 4). Translational elongation factors (EEF1E1 and EEF1A2) were also down-regulated (Figure 5). Previous findings have indicated a reduction in EEF1A expression in AD patients specifically in the hippocampus (Beckelman et al., 2016). Genes down-regulated in the Neuronal System pathway and Transmission across Chemical Synapses included GABRA1, GABRG2, NCALD, GAD1, and NEFL (Figure 5). GABRA1 and GABRG2 are receptors in the gamma-aminobutyric acid (GABA) signaling system that bind to GABA (inhibitory neurotransmitter) and regulate chloride levels in the brain (Padgett and Slesinger, 2010; Calvo-Flores Guzmán et al., 2018). In AD, the GABA signaling system is dysregulated with changes in GABA expression in the hippocampus (Calvo-Flores Guzmán et al., 2018). NCALD is a calcium sensor that is involved in neuronal calcium signaling (Stelzer et al., 2016; Upadhyay et al., 2019). NEFL makes the protein neurofilament light chain (Nfl), which has recently been investigated as a fluid biomarker for monitoring AD disease progression (Preische et al., 2019). Our results also included down-regulated genes PSMA3, PSMC6, and SEM1 that are part of the proteasome complex (cell cycle progression and DNA damage repair) (Tanaka, 2009; Stelzer et al., 2016; Kolog Gulko et al., 2018) and replication factor protein, RPA3 (needed to stabilize single stranded DNA during DNA replication) (Lin et al., 1996; Stelzer et al., 2016), which are down-regulated in the DNA Replication Pre-Initiation and M/G1 Transition pathways. It has been reported that incomplete DNA replication and irregular cell cycle events, such as abnormal cell cycle reentry by neurons have been observed in AD brains and lead to cell death (Yurov et al., 2011). Additionally, dysregulation of the proteasome complex in AD is supported by the literature (Checler et al., 2000; Salon et al., 2003; Oh et al., 2005; Bonet-Costa et al., 2016). However, the role of the proteasome complex in AD and how it is regulated is still not clearly understood (Bonet-Costa et al., 2016), and merits further consideration.

Reactome pathway analysis on the up-regulated genes resulted in some interesting pathways, such as Extracellular Matrix (ECM) Organization, ECM proteoglycans, Mesenchymal Epithelial Transition (MET) activates PTK2 signaling, MET promotes cell motility, Non-integrin Membrane-ECM interactions and Syndecan Interactions, which all had overlapping genes (Figure 5). CAPN3, COL21A1, EFEMP2, and ITGB8 were only in the ECM organization pathway (Figure 6). COL21A1 has been described as being necessary for maintaining the integrity of the ECM, and has been previously found to be up-regulated in severe AD (Kong et al., 2009). Additionally, changes in the ECM components and degradation with proteases have previously been found to be associated with plaque formation, which causes brain dysfunction (Dauth et al., 2016; Sethi and Zaia, 2017; Sonbol, 2018). The up-regulated genes in the potassium and Ca_2+_ channel pathways included GNG12, KCNJ2, KCNJ16, and KCNJ10. In general, as potassium channels open to increase potassium in the cells, calcium is decreased by inhibiting the Ca^2+^ gated channels (Padgett and Slesinger, 2010). Increased activity of the potassium channels, especially the voltage-gated channels have been associated with regulating microglia function and priming which in turn leads to increased ROS production in AD (Rangaraju et al., 2015; Thei et al., 2018).

We compared the 352 genes identified as differentially expressed and exhibiting a biological effect with respect to disease status to a recently published meta-analysis in which 1400 differentially expressed disease genes were identified (Moradifard et al., 2018). We determined that 136 DEG from our gene list overlapped with Moradifard et al.'s findings., and 216 of our DEG were not in their list (Moradifard et al., 2018). Genes that were unique to our DEG list included GMPR, ABCA1, NOTCH1 and 2, GABRG1, HVCN1, CXCR4, HIP1, MRPS28, FOS.

The top up-regulated gene in AD from our meta-analysis, ITPKB (Table 4) has previously been observed to have over-expression in AD subjects. In a mouse model, the gene was found to be over-expressed and connected to apoptosis, increased (Aβ) production and tau phosphorylation (Stygelbout et al., 2014). Additional DEG included CXCR4 (brain development and neuronal cell survival in the hippocampus) (Stelzer et al., 2016; Li and Wang, 2017), AHNAK (may have a role in development of neuronal cells) (Gentil et al., 2005; Stelzer et al., 2016), NOTCH1,and NOTCH2 (signaling pathway may be involved in brain development) (Ables et al., 2011; Stelzer et al., 2016) which were all up-regulated in AD subjects (Table 4). On the other hand, RPA3 (DNA replication), NME1 (neural development) (Owlanj et al., 2012; Stelzer et al., 2016), and mitochondrial proteins MRPL3, MRPS18C (associated with mitochondrial dysfunction observed in AD) were down-regulated in AD samples (Table 4).

### 4.2. Sex, Age, and Tissue Effect on Disease Status Biologically Significant Genes

For the sex factor, we determined that 46 of our DEG (23 up- and down-regulated in males compared to females) had a sex effect, with 1 of them (CXCR4) showing a statistically significant (*p*-value < 0.05) interaction between disease status and sex. The enriched pathways from the up-regulated genes (prior to selecting for interacting genes) in males are highlighted in Figure S8. Furthermore, these genes involved in pathways, such as Clathrin-mediated endocytosis (SNAP91, SH3GL2, and AMPH), Neuronal System, Neurotransmitter receptors postsynaptic transmission and Transmission across Chemical Synapses (GABRG2, GABRA1, GAD1, and NEFL) were down-regulated in females (Figure S8 and Table S3). Down-regulation in genes, such as GABRG2, GABRA1, GAD1, and NEFL) was previously discussed as being down-regulated in AD from our DEG list for disease status (Figure 5).

Additionally, the current literature indicates that women are at higher risk for AD (Seshadri et al., 1997; Vina and Lloret, 2010; Podcasy and Epperson, 2016). This increased risk by sex is due to the loss of estrogen protection (due to menopause) against (Aβ)'s toxicity on the mitochondria (Vina and Lloret, 2010; Podcasy and Epperson, 2016). Older women produce more reactive oxygen species with the decline in estrogen levels (Vina and Lloret, 2010; Podcasy and Epperson, 2016). Estrogen replacement therapy is a treatment for AD, and it is being determined that estrogen works by increasing the expression of antioxidant genes (Vina and Lloret, 2010; Podcasy and Epperson, 2016). A recently published meta-analysis also explored sex effects on AD gene expression (Moradifard et al., 2018). Moradifard et al., found male and female specific AD associated genes and genes that overlapped in both sexes (Moradifard et al., 2018). Of the 46 disease associated genes we found to be affected by sex, 22 were found in both males and females, 9 only in males, and 5 only in females in Moradifard et al gene list. Ten of our sex impacted disease genes (CYBRD1, DIRAS2, FAM107B, FOS, GMPR, HVCN1, ITIH5, MAPK, RNF135, SLC40A1) did not overlap with their findings, and these genes have been previously associated with oxidative stress, cell signaling and transport, apoptosis and AD. For instance, GMPR was found to gradually increase as AD progressed (Liu et al., 2018). It produces GMPR1 which is associated with the phosphorylation of tau (Liu et al., 2018).

Focusing on the statistically significant pairwise interaction between disease status and sex, we identified CXCR4 which was up-regulated in females (Table S3). CXCR4 was also up-regulated in AD (Table 4). CXCR4 has been previously investigated for its role in AD and other neurodegenerative diseases (Bezzi et al., 2001; Li and Wang, 2017; Bonham et al., 2018). CXCR4 is a chemokine receptor that binds to CXCL12, and together they are involved in signaling pathways for inflammation and neuronal system function (Bezzi et al., 2001; Li and Wang, 2017; Bonham et al., 2018). CXCR4/CXCL12 together regulate synaptic plasticity, apoptosis, calcium levels, microglia to neuron communication, neuronal signaling and neuroinflammation (Bezzi et al., 2001; Li and Wang, 2017; Bonham et al., 2018). Dysregulation of CXCR4 has been associated with neurodegenerative diseases (Li and Wang, 2017; Bonham et al., 2018). More specifically, up-regulation of CXCR4 in in a mouse model led to abnormal signaling in microglia and tauopathy (Bonham et al., 2018).

Aging trends on the differentially expressed disease genes were visualized in Figure S10 and Figure 7. Subjects grouped as <60 were used as a baseline because on average, AD symptoms start at ages 65 and older (Masters et al., 2015). We observed clear age-related patterns when looking at the difference of means between age cohorts (prior to selecting for interacting genes) for the disease gene list (Figure S10 and see ST10 of online Supplementary Datasheet). Highlighting a few of the changes: SNAP91 which is involved in synaptic transmission and associated with late onset (Zhang et al., 2013), STMN2 which is necessary for microtubule dynamics and neuronal growth (Antonsson et al., 1998; Chiellini et al., 2008), and SST, a neuropeptide that interacts with (Aβ) and can influence how it aggregates (Hama and Saido, 2005; Solarski et al., 2018) were all up-regulated in <60 age group (Figure S10 and see ST8 of online Supplementary Datasheet). Also, STMN2 and SST have both previously been associated with expression reduction due to age(Stelzer et al., 2016; Solarski et al., 2018). ABCA1, GMPR, HVCN1, ITPKB, NOTCH1 all had higher expression in older age groups compared to the baseline.

Furthermore, visualizing the genes with a statistically significant interaction (*p*-value < 0.05) between disease and age group, we observed three distinct groups of genes with similar patterns (Figure 7). Genes identified in group 1 in Figure 7 were down-regulated in ages 65–80 compared to the baseline (<60 years old). Group 1 genes also displayed a slight increase in relative expression from ages 85 and higher (Figure 7). Reactome pathway analysis on the group 1 genes identified 3 enriched pathways that were statistically significant (FDR < 0.05): (i) MECP2 regulates transcription of genes involved in GABA signaling (GAD1) (He et al., 2014; Fabregat et al., 2017), (ii) Muscarinic acetylcholine receptors (CHRM1) (Ishii and Kurachi, 2006; Fabregat et al., 2017) and (iii) Neuronal System (CACNG3, GAD1, NEFL, GABRA1, GLRB, NRXN3, GABRG2, and KCNQ2) (Purves D, 2001; Fabregat et al., 2017). Changes in GABA signaling in AD was previously characterized as age-dependent (Limon et al., 2012). The ionic response to GABA, also reported as GABA currents, were reduced in AD, especially in younger subjects with AD (Limon et al., 2012). We observe a similar pattern in our meta-analysis for the GABA receptor genes in group 1 (Figure 7). Genes within group 2 displayed a gradual increase in expression with age (Figure 7). Reactome pathway analysis did not identify statistically significant enrichment for these genes. However, genes in group 2 include DDR2 (regulates TREM2, microglia and neurotoxic proteins) (Hebron et al., 2017), IP6K3 (Inositol phosphate metabolism) (Crocco et al., 2016), and GJA1 (regulates known AD risk factor genes) (Kajiwara et al., 2018). Additionally, genes in group 3 exhibited significant up-regulation in gene expression for subjects 65–80 years with a gradual decrease in expression from ages 85 and older (Figure 7). These genes are associated with the statistically significant enriched pathway (FDR < 0.05), TRAF6 mediated NF-kB activation (MAP3K1) (Yoshida et al., 2008; Fabregat et al., 2017). Our findings highlight genes previously associated with AD and their temporal trends, and also some additional genes that experience age-effects (Figure 7 and Figure S10, and see ST10 of online Supplementary Datasheet).

To investigate tissue-specific effects (prior to selecting for statistically significant pairwise interactions between tissue and disease status), we used hippocampus (232 samples) as a baseline due to it being identified as one of the first regions to be affected by AD (Masters et al., 2015). We also used blood (519 samples) as a baseline to explore an underdeveloped non-invasive approach to monitoring AD. In both analyses, we saw similar trends with the nucleus accumbens (51 samples) and putamen (52 samples) showing greater differences in expression (Figures S11, 12). Focusing on the genes that showed a statistically significant interaction between disease and tissue, we observed lower expression of genes in tissues compared to the hippocampus and blood with a slight increase in the primary visual cortex and the putamen (Figure 8). As for the nucleus accumbens we observed significantly higher expression for these interacting genes for both hippocampus and blood baseline comparisons (Figure 8). The statistically significant (*p*-value < 0.05) interacting genes in Figure 8 include genes that are involved in development of dendritic spines (C21orf91), normal brain function (SELENOP), GABA signaling (GABRG1), and structure of actin cytoskeleton (EPS8) (Menna et al., 2015; Pitts et al., 2015; Li et al., 2016; Stelzer et al., 2016; Calvo-Flores Guzmán et al., 2018). In addition to the shrinking of the hippocampus, decreases in volumes for nucleus accumbens and the putamen have also been reported (de Jong et al., 2008; Nie et al., 2017). The nucleus accumbens is important for reward processing, and in AD has been associated to impaired decision making and reduction in performance of rewarding behaviors (Nobili et al., 2017). AD is also associated with reduced dopamine levels and GABA signaling (Martorana and Koch, 2014). Finally, the putamen (motor behaviors) and primary visual cortex (visual processing) both have impaired functions in AD (Halabi et al., 2013; Brewer and Barton, 2014).

The distribution of samples per tissue type was inconsistent with hippocampus and blood having larger number of samples compared to an average of around 55 samples per tissue in other categories. These results show the potential of blood and other tissues for monitoring gene expression changes in AD, but also the need for further focused mechanistic studies in different tissues.

### 4.3. Limitations of the Study

Using publicly available data introduced limitations to our research design. Lack of uniform annotation and missing information across datasets can make conducting a meta-analysis on multiple datasets challenging. For example the subclass of AD, details on cognitive status and APOE genotype were not uniformly reported across the datasets used (Table S1). The brain samples were from a variety of brain banks with varying institutional review boards and standards, protocols and criteria for AD diagnosis requirements (Table S1). Additionally, the number of datasets used in our meta-analysis was limited by poor annotations that could not meet our selection criteria, and this in turn placed bounds to our sample size and power of the study. Our analysis was also unbalanced: 2,088 samples made up of 771 healthy controls, 868 AD subjects, 449 subjects reported as possibly having AD, 1308 females and 780 males, and the breakdown of age groups is also somewhat uneven. One of our datasets (GSE84422) consisted of paired samples. However, as the the other datasets did not include paired samples, we did not incorporate a paired-sample analysis in our study. The available public data used for our meta-analysis also lacked diversity in samples, because in most datasets race and ethnicity are not reported. This information would be helpful particularly since AD has been reported by the CDC to be more prevalent in African Americans (Steenland et al., 2016; Centers for Disease Control and Prevention, 2018). In addition, the use of micro-array expression data for meta-analysis is a limitation in terms of not being able to query the entire transcriptome or query novel genes. Also, in our merged dataset, large variability was introduced in data due to the large number of tissues (26) and methods used for extractions (study effect), which we attempted to correct for by utilizing both as factors in our model, and including binary interaction terms as well. An additional limitation of our study is that we included datasets that investigated gene expression changes in bulk tissue rather than on the cell-type-specific level. Cell-type-specific expression data that matched our inclusion criteria were not available to include in this meta-analysis. Furthermore, single-cell data is also only recently becoming available. A meta-analysis including single-cell analysis expression data from specific cell types, such as neurons, astrocytes and microglia would allow an improved understanding of gene expression differences between AD and healthy controls (Wang and Bodovitz, 2010; Stuart and Satija, 2019). Finally, to our knowledge, there were also a limited number of RNA-sequencing (RNA-seq) datasets on GEO and Array Express (23), and only one that matched our selection criteria. Thus, we elected to carry out the analysis using the gene expression array data. We anticipate that more RNA-seq data, which can provide a more global view of the transcriptome, will become available in the future.

### 4.4. Future Directions and Recommendations

Our study provides gene lists by factor (disease status, sex, age, and tissue) of differentially expressed genes. Our study is largely descriptive, but also yields new gene candidates which we may be studied further for their role in AD, including underlying mechanisms using model systems. To expand on this research, the use of RNA-seq data can reveal novel differentially expressed genes, biomarkers and gene targets for AD. As more RNA-seq data becomes available, a similar meta-analysis approach may be applied, if such data are annotated to include the necessary factors' metadata for the analysis. In addition to RNA-seq, implementing other omics technologies, such as proteomics and metabolomics can help to fully describe the pathology of AD, and identify additional biomarkers for early detection. To promote more meta-analyses, we recommend that future studies include more extensive, and structured standardized metadata in their submissions, that will enable use of data. Including data with racial diversity is also necessary. AD has higher prevalence in African Americans (Steenland et al., 2016). Due to reports of racial differences in AD, with an AD prevalence breakdown of: 14% of African American population compared to 12% in Hispanics and 10% in whites (Centers for Disease Control and Prevention, 2018), including racial diversity in future studies would help identify this potential variability in susceptibility and identify if certain treatments might be better suited in some races than others. Improving the representation of races in clinical trials and molecular reports of AD can help with health disparities within the field. Exploring the use of easily accessible tissues, such as blood, to monitor changes in target genes/biomarkers might also prove helpful for early detection and provide a more systems-level understanding of AD. Determining the best or novel biomarkers to track for AD requires exploring also mechanistic aspects of the disease. For example, monitoring exosomes and autoantibodies which can be connected to the dysfunction of the immune system is one mode of action that is being associated with AD (O'Bryant, 2016). Lastly, as omics technologies advance, implementing personalized omics for early detection and treatment may prove useful in improving individual AD outcomes with the increase in the aging population.

## Author Contributions

All authors listed have made a substantial, direct and intellectual contribution to the work, and approved it for publication.

### Conflict of Interest Statement

GM has consulted for Colgate-Palmolive. LB declares the absence of any commercial or financial relationships that could be construed as a potential conflict of interest.

## Supplementary Material

Supplementary Datasheet Our supplemental figures and tables are provided in the accompanying supplementary data pdf file. All of our datasets/results from our meta-analysis pipeline have been uploaded to FigShare as online supplemental data, as described below (the corresponding online file names begin with a prefix “ST” and are enumerated as also referred to in the manuscript). The datasets generated and analyzed for this study can be found in Figshare's online digital repository at https://doi.org/10.6084/m9.figshare.7435469.
