# Supplementary material for: Data-Driven Analysis of Age, Sex, and Tissue Effects on Gene Expression Variability in Alzheimer's Disease
Source: Front Neurosci. 2019 Apr 24;13:392. doi: 10.3389/fnins.2019.00392 (PMC6491842; doi:10.3389/fnins.2019.00392)
Supplement: Supplementary Datasheet — Our supplemental figures and tables are provided in the accompanying supplementary data pdf file. All of our datasets/results from our meta-analysis pipeline have been uploaded to FigShare as online supplemental data, as described below (the corresponding online file names begin with a prefix “ST” and are enumerated as also referred to in the manuscript). The datasets generated and analyzed for this study can be found in Figshare's online digital repository at https://doi.org/10.6084/m9.figshare.7435469. [file Data_Sheet_1.pdf]

Supplementary Material: Data-Driven Analysis of Age, Sex, and Tissue Effects on Gene Expression Variability in Alzheimer's Disease

1 SUPPLEMENTARY TABLES AND FIGURES

1.1 Tables

Dataset	Cognitive Data Reported	Additional Notes	Brain Bank
**GSE84422**	• Braak stage<br>• Neuropathological category<br>• Clinical dementia rating<br>• CERAD scores<br>• Sum of neurofibrillary tangles density<br>• Average plaque density	• **AD: Probable/possible/definite**<br>• Post-mortem<br>• APOE genotype not reported<br>• Full spectrum of clinical and neuropathological disease severity<br>• Excluded subjects with non-AD neuropathology<br>• Mount Sinai and JJ Peters Institutional Review Boards approved protocols	Mount Sinai Medical Center Brain Bank
**GSE28146**	• Mini-mental state examination<br>• Braak stage (averaged)<br>• Neurofibrillary tangle density (averaged)	• **AD: Incipient/moderate/severe**<br>• Post-mortem<br>• APOE genotype not reported<br>• Alzheimer's Disease and Related Disorders Association criteria	Brain Bank of Alzheimer's Disease Research Center at the University of Kentucky
**GSE48350**	• Braak stage<br>• Mini-mental state examination	• Post-mortem<br>• APOE genotype reported<br>• Excluded subjects with evidence of alcoholism, co-existing major psychiatric illness or major depression, pre-existing brain damage, brain metastases and cerebral vascular disease<br>• Excluded subjects with non-AD neuropathology	National Institute on Aging Alzheimer's Disease brain banks
**GSE5281**	• Braak stage (range provided, not reported per sample)<br>• CERAD scores (range provided, not reported per sample)	• **AD: Late-onset AD**<br>• Post-mortem<br>• APOE genotype not reported<br>• Clinically and neuropathologically classified late-onset AD-afflicted individuals<br>• Braak stage of V or VI	Sun Health Research Institute and Alzheimer's Disease Center at Washington University and Duke University
**GSE63060-1**	• Clinical dementia rating and sum of boxes score (averaged)<br>• Mini-mental state examination	• Living volunteers<br>• APOE genotype not reported<br>• Ethical approval received from Institutional Research Ethics Committee	Not applicable

Table S1. Additional information reported from datasets on samples used for the meta-analysis.

Dataset	Cognitive Data Reported	Additional Notes	Brain Bank
**GSE29378**	• Braak Stage reported for some samples<br>• Plaque disease burden<br>• Disease Duration	• **AD:Late-onset AD**<br>• Post-mortem<br>• APOE genotype reported for some samples<br>• National Institute for Neurological and Communicative Disorders and Stroke-Alzheimer's Disease and Related Disorder Association diagnostic criteria for clinical AD<br>• Neuropathologic confirmation at autopsy	Alzheimer's Disease Center, Oregon Health and Sciences University and Human Brain and Spinal Fluid Resource Center
**E-MEXP-2280**	• Braak Stage reported for some samples<br>• MAPT haplotype	• **AD: Braak stage VI**<br>• Post-mortem<br>• APOE genotype reported<br>• All patients were screened for Microtubule Associated Protein Tau (MAPT) and Progranulin (GRN) mutations and MAPT haplotyping	Netherlands Brain Bank

Table S1 Continued. Additional information reported from datasets on samples used for the meta-analysis.

Quantile	Disease (control-AD)	Sex (male-female)	AgeGroup (i-<60)	Tissue (i-blood)	Tissue (i-hippocamppus)
0.1%	-0.1980181	-0.1850368	-1.8342734	-1.4193659	-1.2176260
1%	-0.1409218	-0.1662240	-1.5978577	-1.1791001	-0.8806144
2.5%	-0.1242022	-0.1531487	-1.4093136	-0.9535290	-0.6994610
5%	-0.1109185	-0.1286464	-1.2380410	-0.7905619	-0.5988491
**10%**	**-0.0944796**	**-0.0863796**	**-1.0477827**	**-0.6359497**	**-0.5187091**
**90%**	**0.1195751**	**0.2502144**	**0.3308682**	**0.7932871**	**0.8181017**
95%	0.1398357	0.2678312	0.4815650	1.0342074	1.0113406
97.5%	0.1597702	0.2788782	0.6502154	1.2459840	1.2049823
99%	0.1851621	0.3036726	0.8852537	1.5229805	1.6578388
99.9%	0.2625072	0.3698125	1.1441852	1.7230551	1.7531744

Table S2. Quantiles on differences of means between group comparisons from TukeyHSD analysis for each factor with the 10% and 90% highlighted.

**Gene**	diff	lwr	upr	tukey.p.adj
SNAP91	0.38789336	0.3162492	0.45953751	<5.91E-12
AMPH	0.261109	0.227946	0.29427199	5.91E-12
CABP1	0.25221566	0.2197738	0.28465749	5.91E-12
CCK	0.2736884	0.2290262	0.31835057	5.91E-12
CHGB	0.27223361	0.2330743	0.31139295	5.91E-12
CPQ	-0.15134075	-0.1739617	-0.12871979	5.91E-12
CXCR4	-0.18569224	-0.2153576	-0.15602687	5.91E-12
DIRAS2	0.26469562	0.2210822	0.30830901	5.91E-12
EEF1A2	0.29830496	0.2546981	0.34191185	5.91E-12
GABRG2	0.28727303	0.2421887	0.33235734	5.91E-12
GFAP	0.26148848	0.2372962	0.28568071	5.91E-12
GJA1	0.30536761	0.279178	0.33155722	5.91E-12
KLF2	-0.16010858	-0.1822269	-0.13799028	5.91E-12
MYT1L	0.25975404	0.2232677	0.29624039	5.91E-12
NEFL	0.27515335	0.2353672	0.31493946	5.91E-12
NRN1	0.26422817	0.2300824	0.29837391	5.91E-12
RGS4	0.27860758	0.2432385	0.31397667	5.91E-12
SERPINI1	0.26217204	0.2301731	0.29417102	5.91E-12
SH3GL2	0.30717515	0.2681965	0.34615382	5.91E-12
IL13RA1	-0.15586061	-0.1816785	-0.13004272	5.91E-12
ERC2	0.26822985	0.2230493	0.31341042	5.91E-12
GAD1	0.26177649	0.2178336	0.30571939	5.91E-12
SLC40A1	-0.18276614	-0.2136628	-0.15186946	5.91E-12
ITIH5	-0.16639611	-0.1947932	-0.137999	5.91E-12
FAM19A1	0.269102	0.2230286	0.31517537	5.91E-12
FGF13	0.25310759	0.2088214	0.29739382	5.92E-12
AHNAK	-0.10311728	-0.1242383	-0.08199628	5.93E-12
RPA3	-0.13337106	-0.1607507	-0.10599143	5.93E-12
EZR	-0.1182311	-0.141882	-0.09458025	5.93E-12
ITPKB	-0.11649742	-0.1417882	-0.09120668	5.93E-12
GABRA1	0.27928408	0.2277225	0.33084567	5.93E-12
MAP3K1	-0.16567888	-0.1964814	-0.13487641	5.93E-12
NOTCH1	-0.10639043	-0.1270303	-0.0857506	5.93E-12
HVCN1	-0.10966873	-0.1333269	-0.0860106	5.93E-12
PCDH8	0.26623656	0.2038808	0.32859232	5.93E-12
LDLRAP1	-0.13026125	-0.1611422	-0.09938027	5.93E-12
GMPR	-0.14621751	-0.1812927	-0.11114232	5.94E-12
CYBRD1	-0.1288122	-0.1605469	-0.09707747	5.94E-12
PRKD2	-0.09889483	-0.1237886	-0.07400105	5.94E-12
PRKX	-0.12798343	-0.1609683	-0.09499854	5.97E-12
STMN2	0.25735011	0.1839117	0.33078852	8.42E-12
HIP1	-0.11198115	-0.1436469	-0.08031542	1.16E-11
FOS	-0.15132275	-0.1952695	-0.10737604	2.55E-11
FAM107B	-0.10351231	-0.1344653	-0.07255936	7.72E-11
RNF135	-0.0875083	-0.1183122	-0.05670445	2.92E-08
ID3	-0.10925012	-0.1502501	-0.06825012	1.94E-07

Table S3. TukeyHSD results (male-female) table of statistically significant differentially expressed disease genes with sex effect.

1.2 Figures

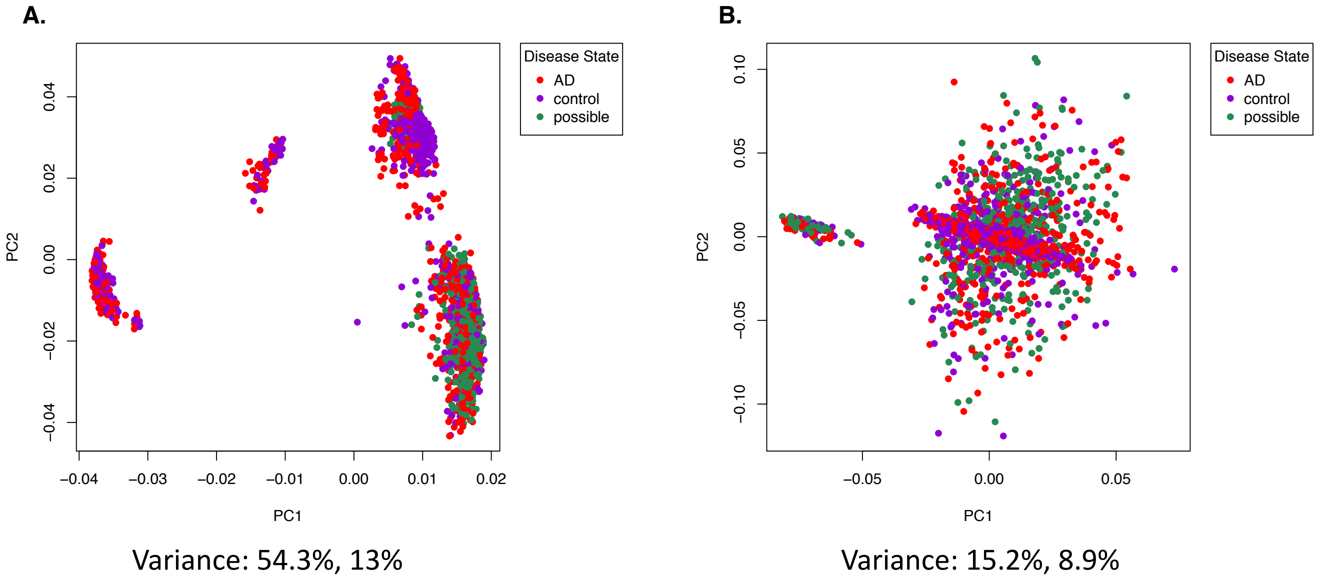

Figure S1. Principal component analysis of the disease factor before (A) and after (B) batch correction with ComBat.

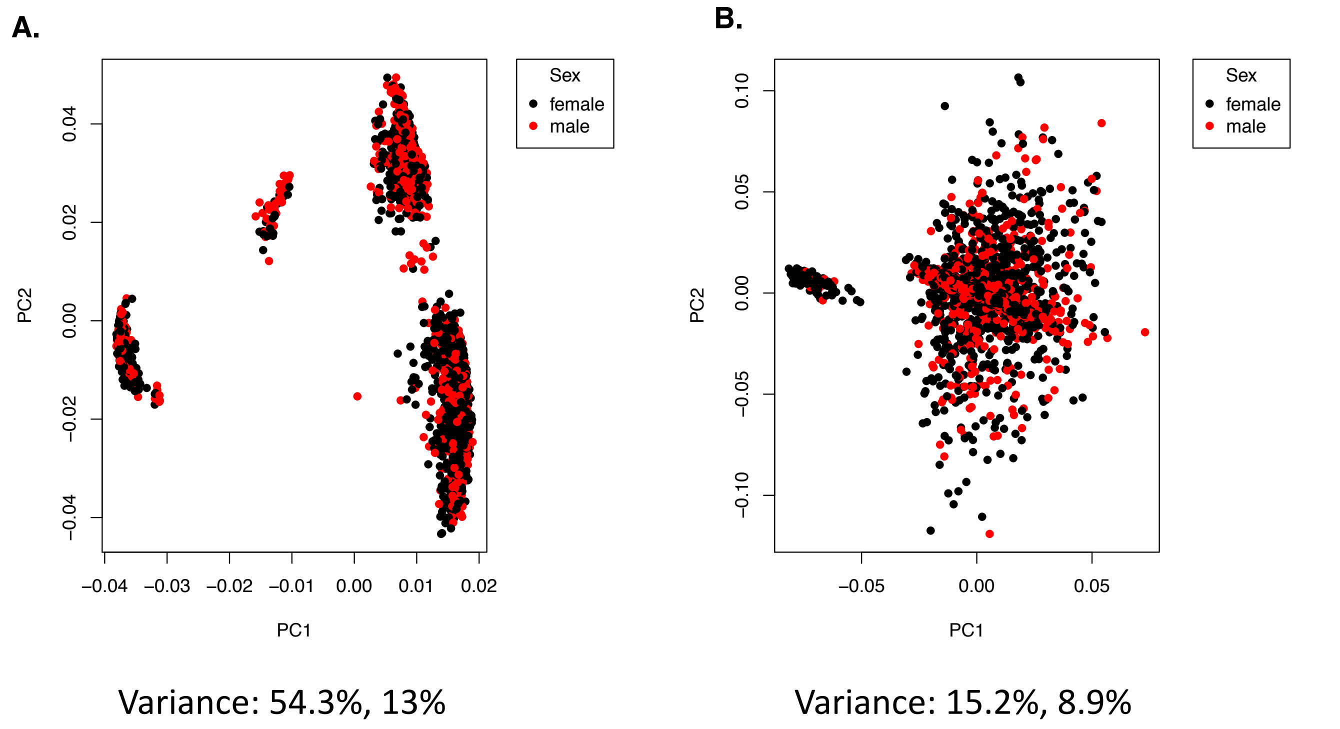

Figure S2. Principal component analysis of the sex factor before (A) and after (B) batch effect correction with ComBat.

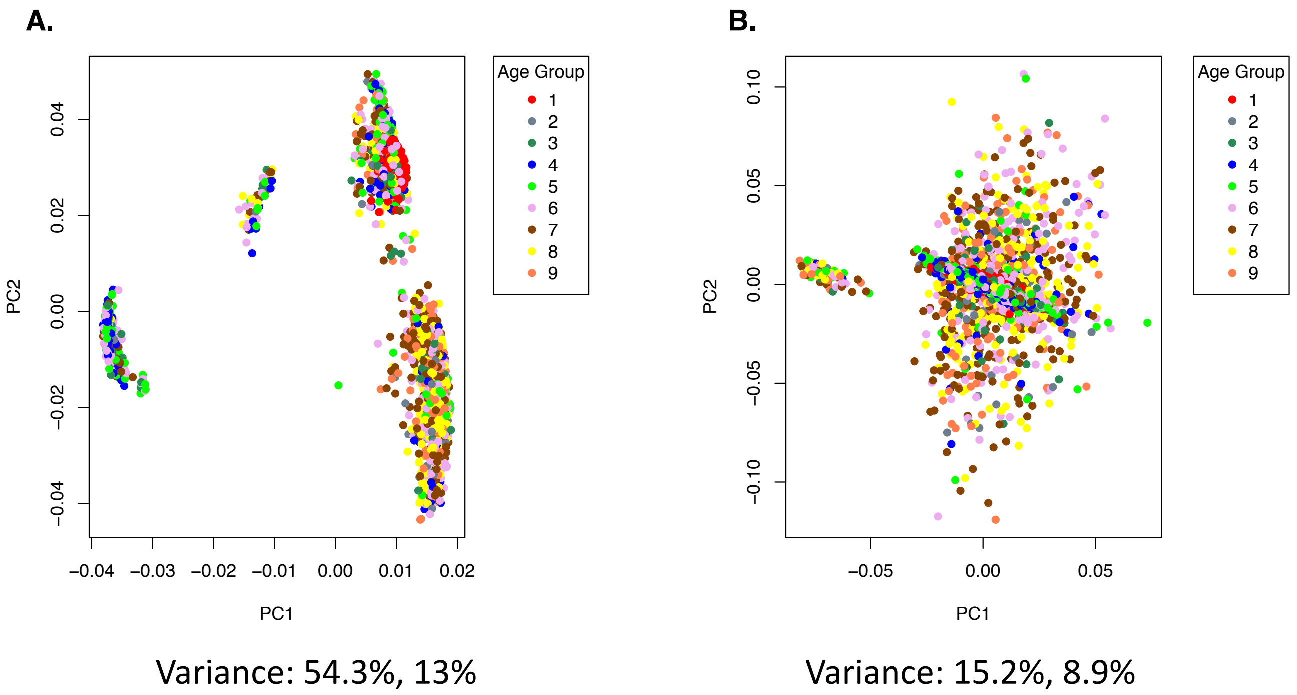

Figure S3. Principal component analysis of the age group factor before (A) and after (B) batch effect correction with ComBat.

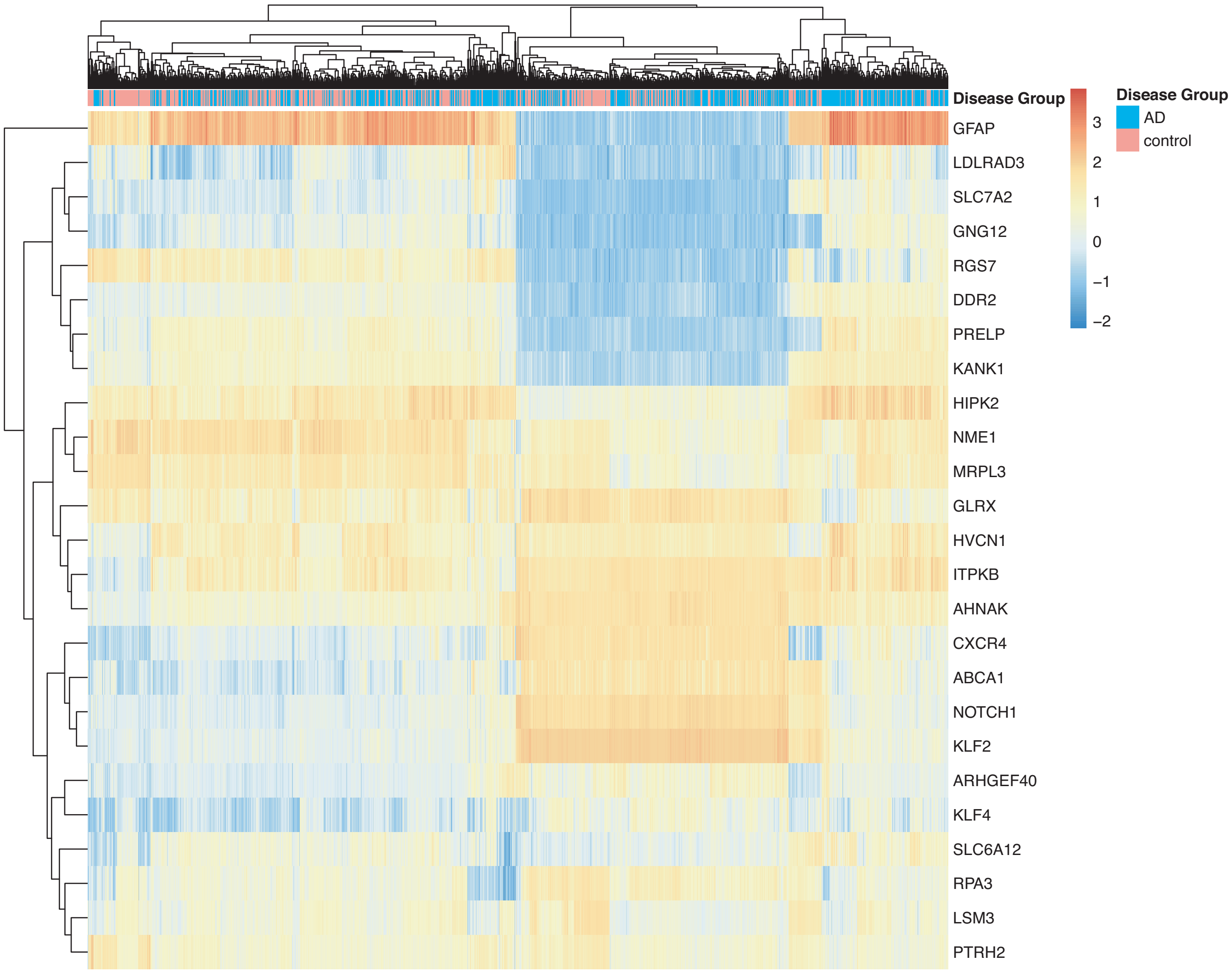

Figure S4. Heatmap with gene clustering of the top 25 differentially expressed disease (control-AD) gene list.

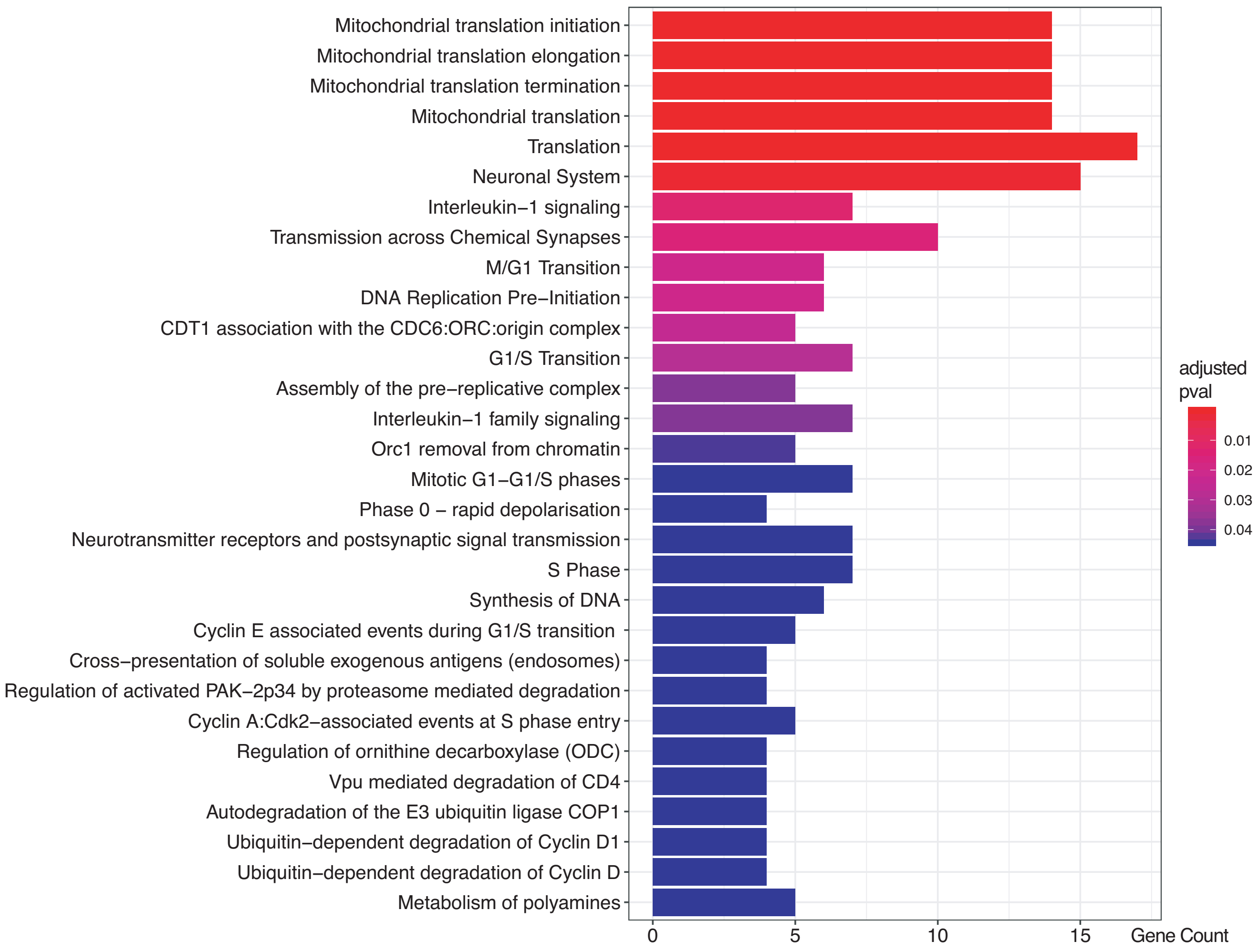

Figure S5. Reactome pathway analysis bar plot of enriched pathways and number of gene hits. Gene list: Genes that were down-regulated in Alzheimer's disease but up-regulated in healthy controls.

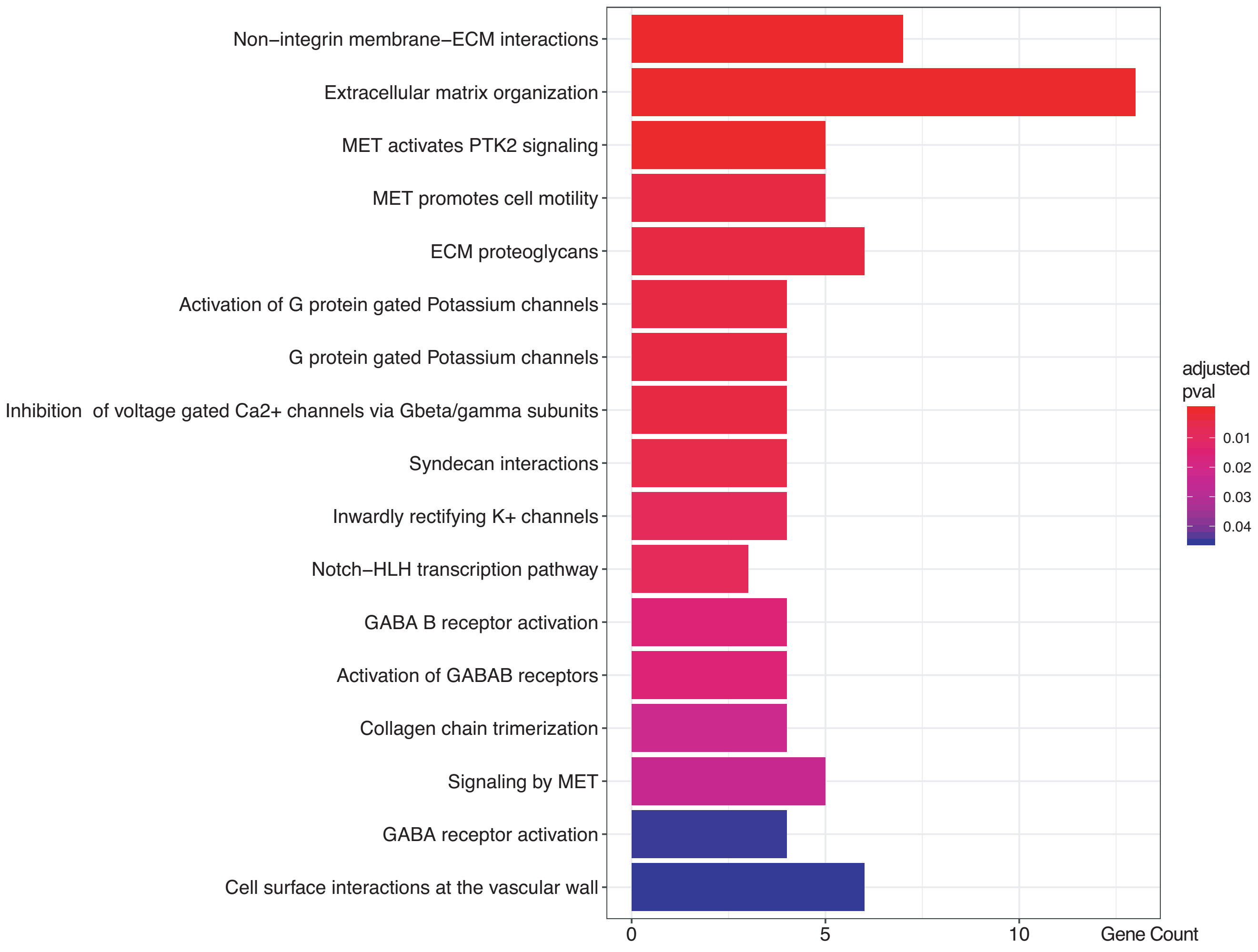

Figure S6. Reactome pathway analysis bar plot of enriched pathways and number of gene hits. Gene list: Genes that were up-regulated in Alzheimer's disease but down-regulated in healthy controls.

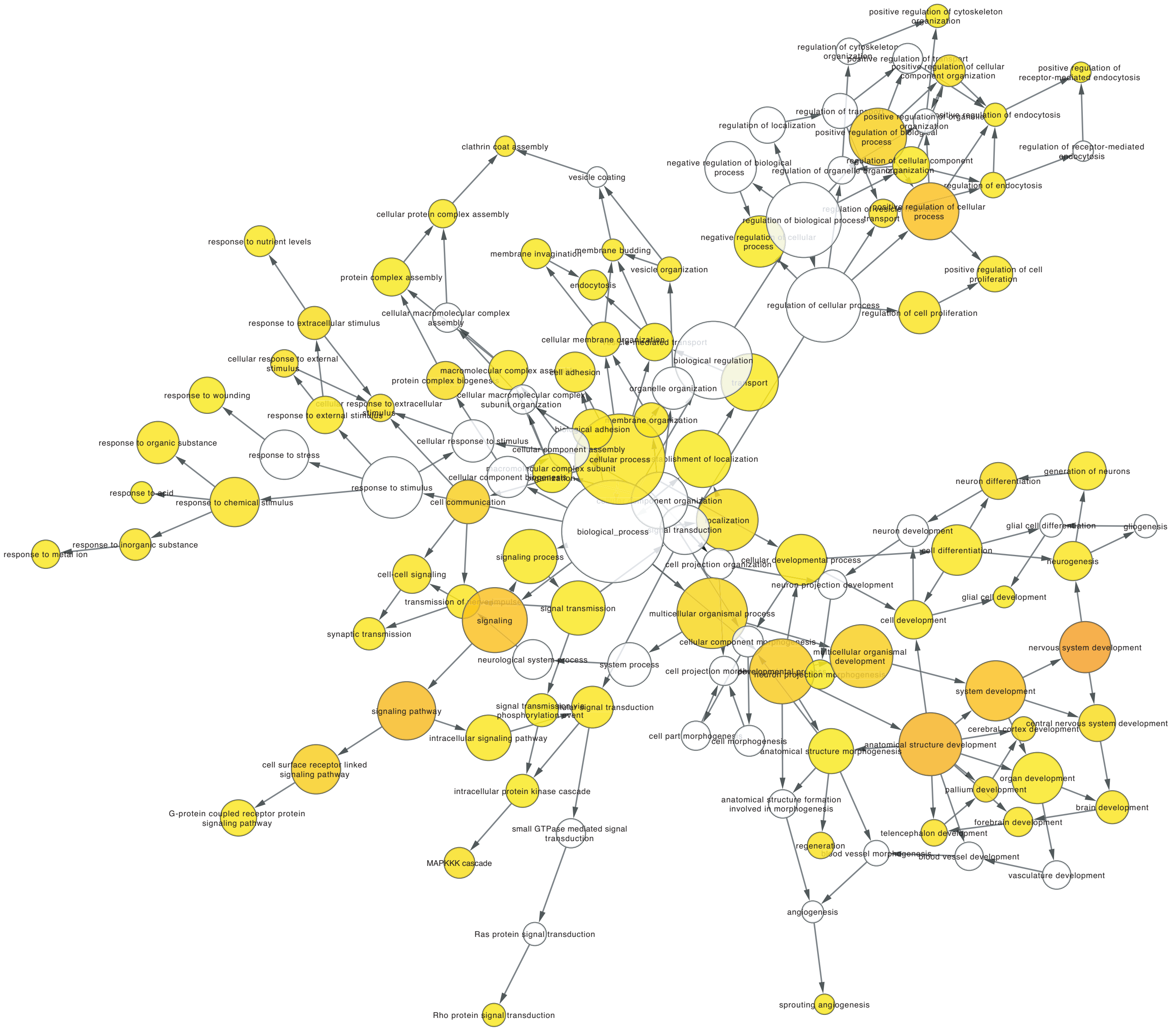

Figure S7. Gene Ontology (biological processes) network of differentially expressed genes by disease factor from BINGO in Cytoscape. The node size relates to number of genes, and the yellow nodes are statistically significant with a p-value < 0.05 and false discovery rate < 0.05.

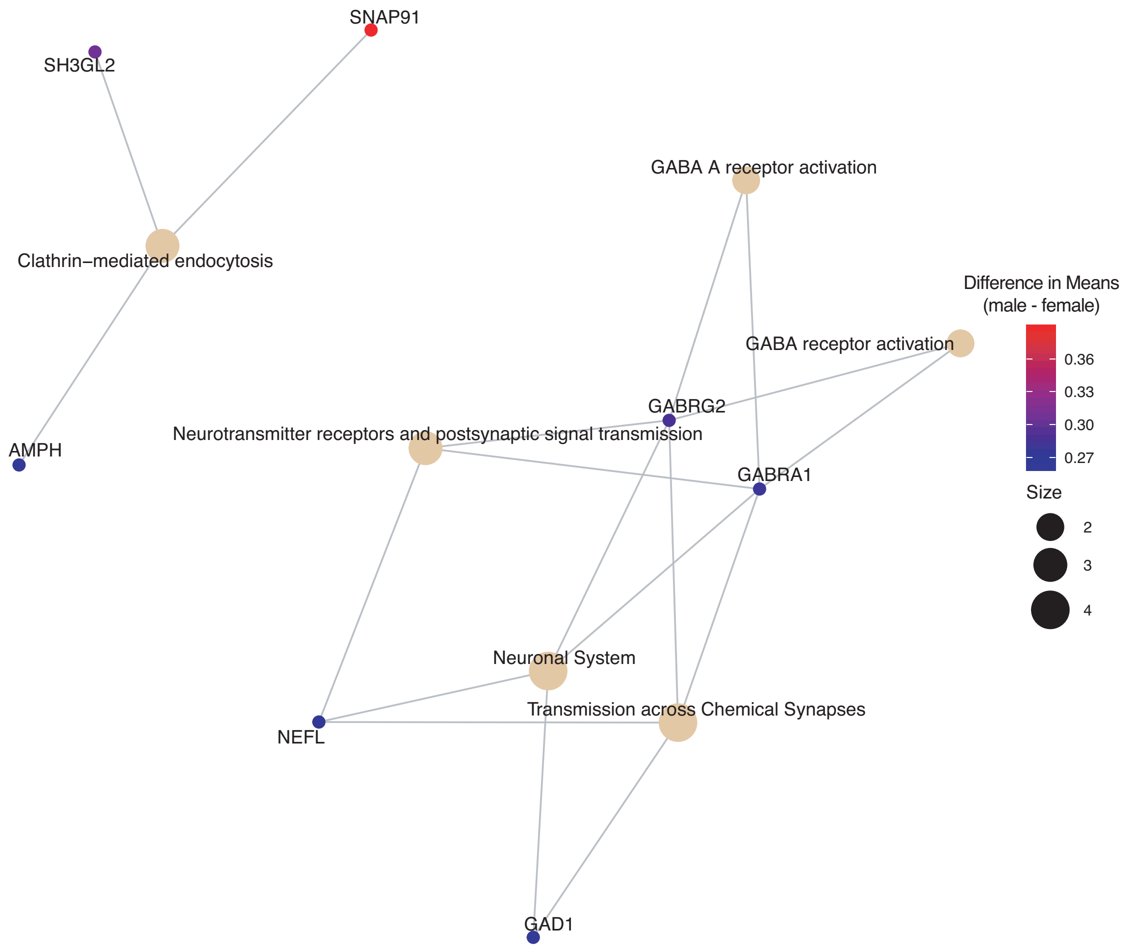

Figure S8. Pathway-gene network of enriched Reactome pathways using the differentially expressed disease genes with a sex effect (prior to selecting for interacting genes) that were up-regulated in males

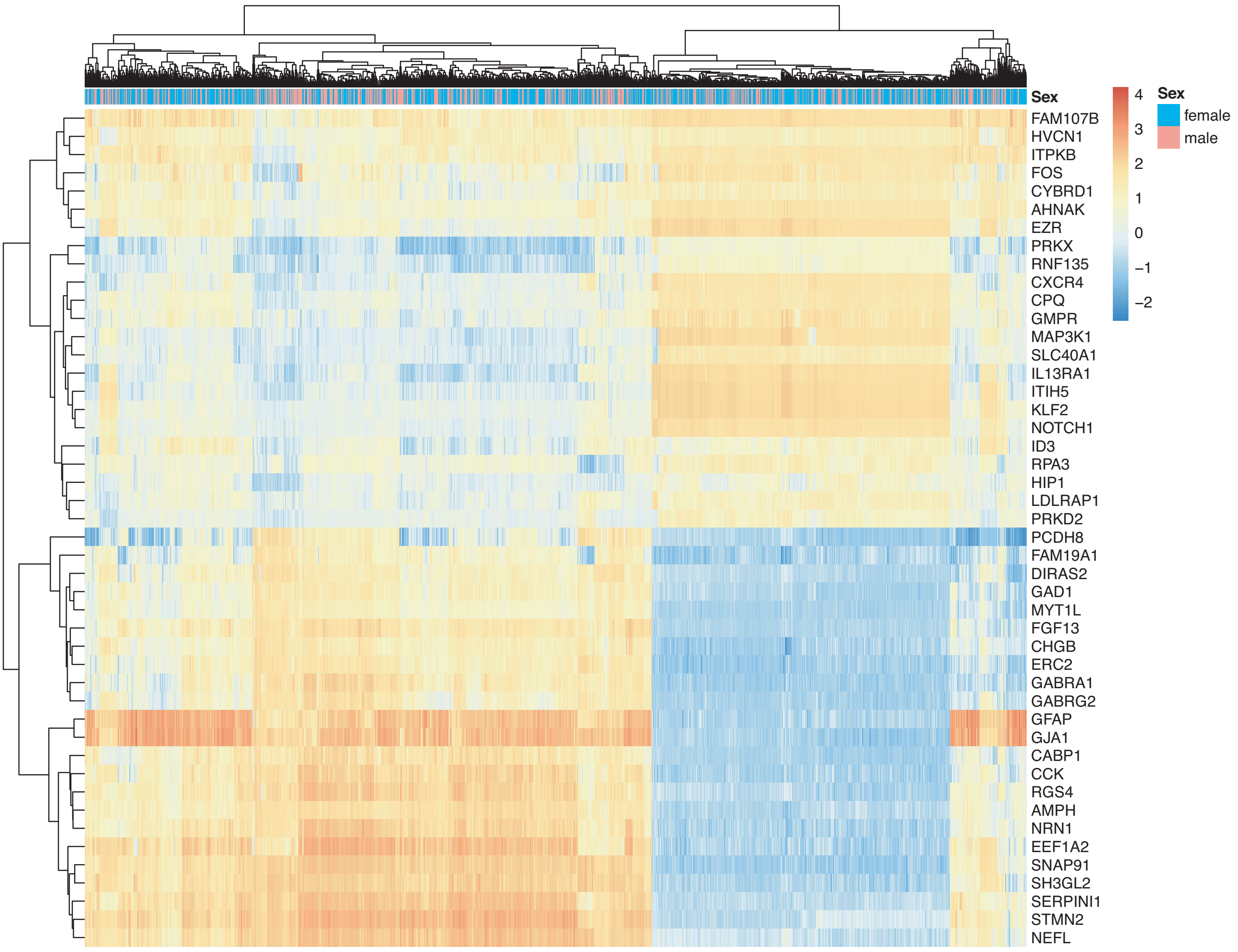

Figure S9. Heatmap with gene clustering to visualize gene expression of differentially expressed disease (control-AD) gene list with a sex effect (prior to selecting for interacting genes).

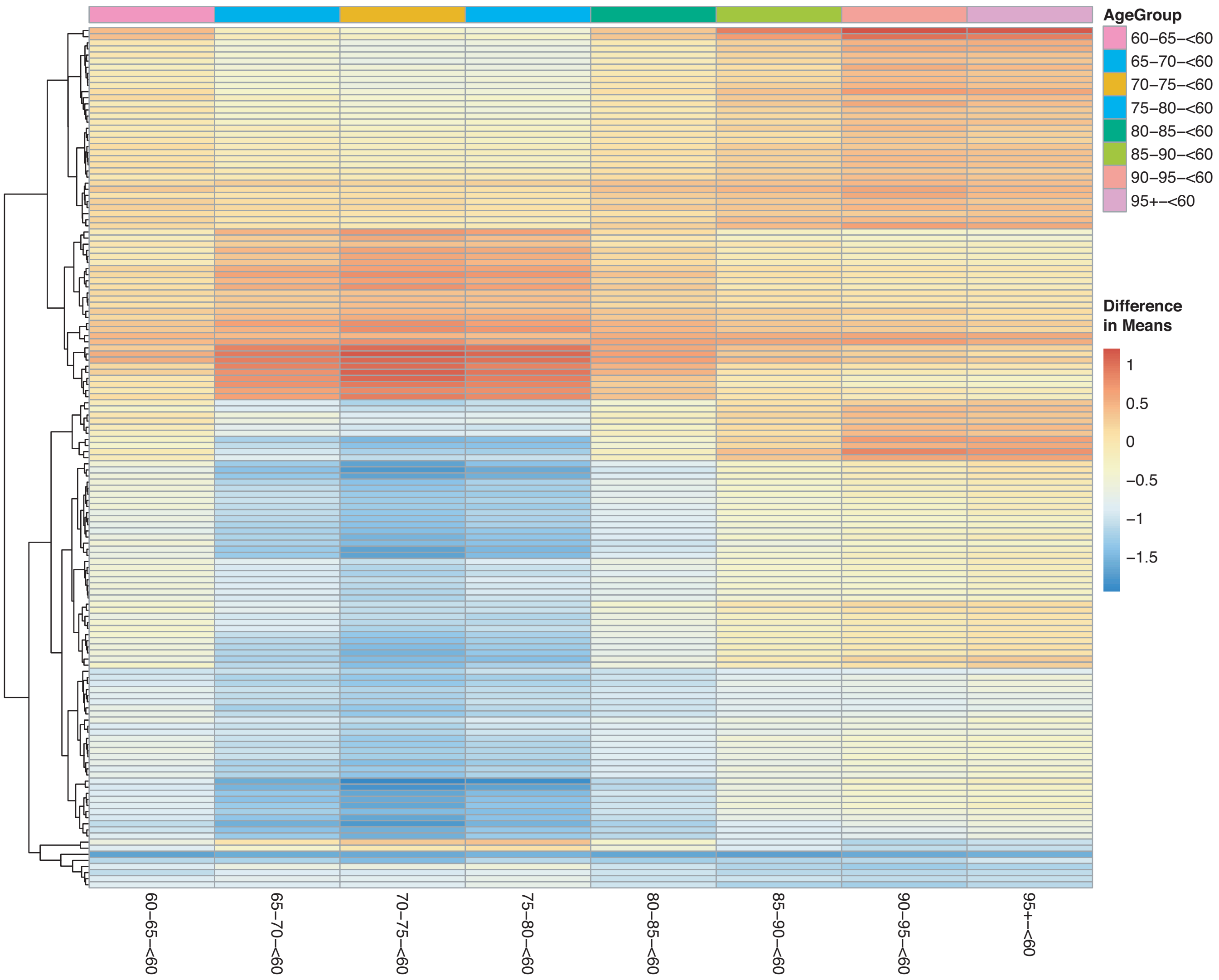

Figure S10. Heatmap with gene clustering to visualize age group effect (prior to selecting for interacting genes) using difference in means on the differentially expressed disease (control-AD) gene list.

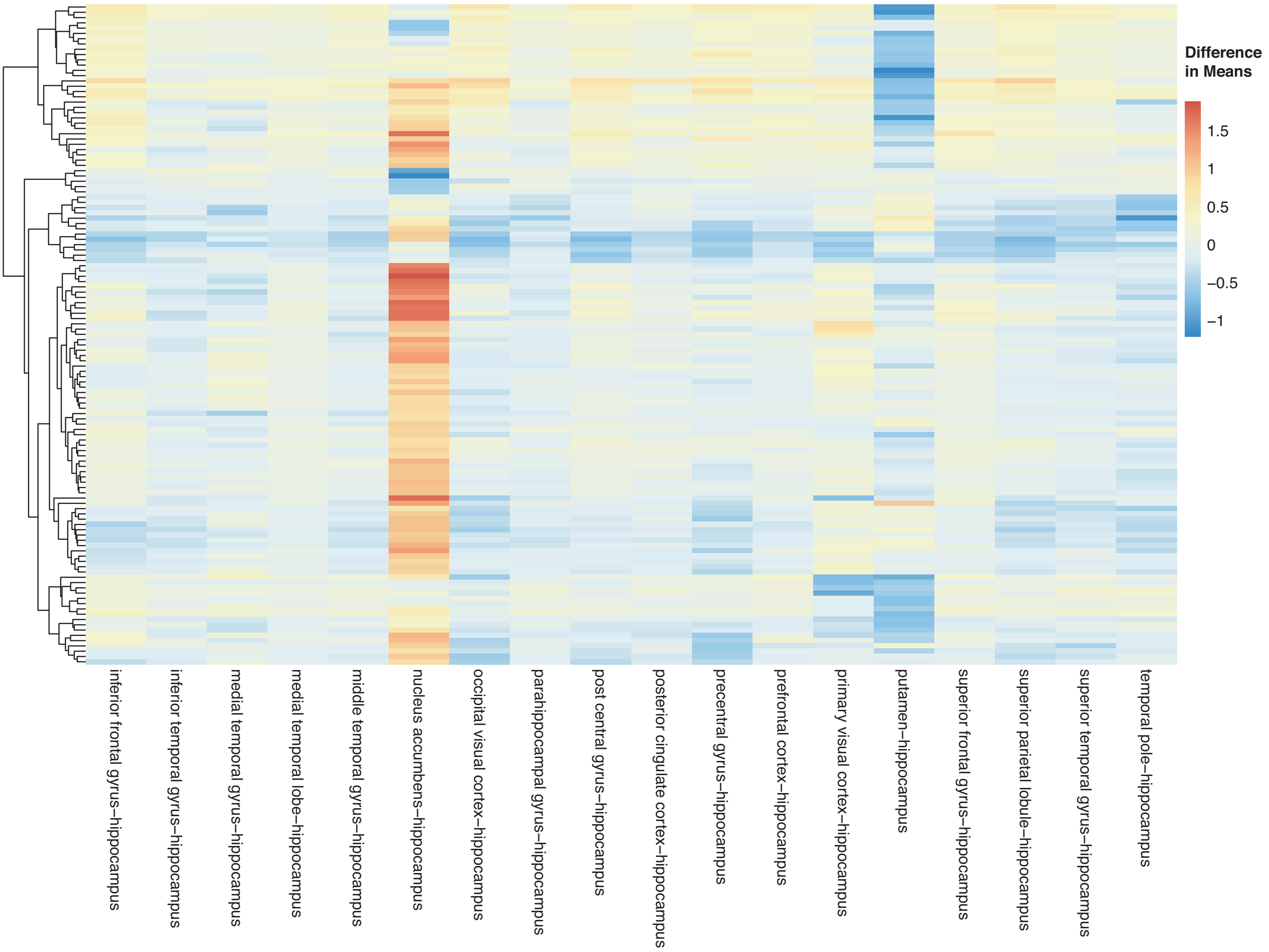

Figure S11. Heatmap with gene clustering to visualize tissue (hippocampus as baseline) effect using the difference in means (prior to selecting for interacting genes) on the differentially expressed disease (control-AD) gene list.

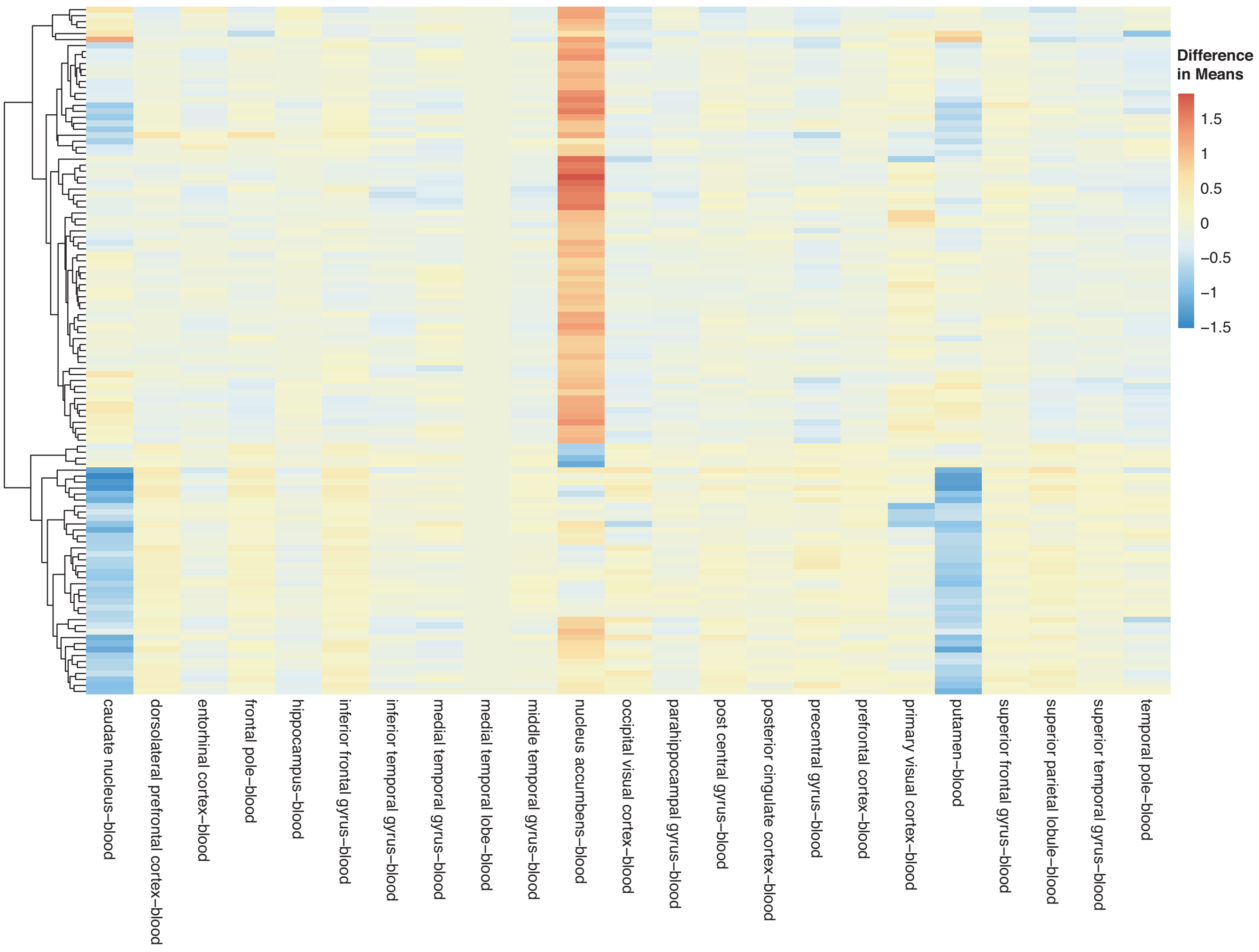

Figure S12. Heatmap with gene clustering to visualize tissue (blood as baseline) effect using the differences in means between binary comparisons (prior to selecting for interacting genes) on the differentially expressed disease (control-AD) gene list .
